# Evaluating the Effects of Opioid Prescribing Policies on Patient Outcomes in a Safety-net Primary Care Clinic

**DOI:** 10.1007/s11606-021-06920-4

**Published:** 2021-06-25

**Authors:** Christopher L. Rowe, Kellene Eagen, Jennifer Ahern, Mark Faul, Alan Hubbard, Phillip Coffin

**Affiliations:** 1 Division of Epidemiology, School of Public Health, University of California, Berkeley, Berkeley, CA USA; 2 Center on Substance Use and Health, San Francisco Department of Public Health, San Francisco, USA; 3 Department of Family Medicine and Community Health, School of Medicine and Public Health, University of Wisconsin-Madison, Madison, USA; 4 Health Systems and Trauma Systems Branch, Centers for Disease Control and Prevention, Atlanta, USA; 5 Division of Biostatistics, School of Public Health, University of California, Berkeley, Berkeley, USA; 6 Division of HIV, Infectious Disease & Global Medicine, University of California San Francisco, San Francisco, USA

**Keywords:** opioid prescribing, primary care, illicit opioid use

## Abstract

**Background:**

After decades of liberal opioid prescribing, multiple efforts have been made to reduce reliance upon opioids in clinical care. Little is known about the effects of opioid prescribing policies on outcomes beyond opioid prescribing.

**Objective:**

To evaluate the combined effects of multiple opioid prescribing policies implemented in a safety-net primary care clinic in San Francisco, CA, in 2013–2014.

**Design:**

Retrospective cohort study and conditional difference-in-differences analysis of nonrandomized clinic-level policies.

**Patients:**

273 patients prescribed opioids for chronic non-cancer pain in 2013 at either the treated (n=151) or control clinic (n=122) recruited and interviewed in 2017–2018.

**Interventions:**

Policies establishing standard protocols for dispensing opioid refills and conducting urine toxicology testing, and a new committee facilitating opioid treatment decisions for complex patient cases.

**Main Measures:**

Opioid prescription (active prescription, mean dose in morphine milligram equivalents [MME]) from electronic medical charts, and heroin and opioid analgesics not prescribed to the patient (any use, use frequency) from a retrospective interview.

**Key Results:**

The interventions were associated with a reduction in mean prescribed opioid dose in the first three post-policy years (year 1 conditional difference-in-differences estimate: −52.0 MME [95% confidence interval: −109.9, −10.6]; year 2: −106.2 MME [−195.0, −34.6]; year 3: −98.6 MME [−198.7, −23.9]; year 4: −72.6 MME [−160.4, 3.6]). Estimates suggest a possible positive association between the interventions and non-prescribed opioid analgesic use (year 3: 5.2 absolute percentage points [−0.1, 11.2]) and use frequency (year 3: 0.21 ordinal frequency scale points [0.00, 0.47]) in the third post-policy year.

**Conclusions:**

Clinic-level opioid prescribing policies were associated with reduced dose, although the control clinic achieved similar reductions by the fourth post-policy year, and the policies may have been associated with increased non-prescribed opioid analgesic use. Clinicians should balance the urgency to reduce opioid prescribing with potential harms from rapid change.

**Supplementary Information:**

The online version contains supplementary material available at 10.1007/s11606-021-06920-4.

## INTRODUCTION

The USA continues to grapple with an unprecedented opioid overdose epidemic. Almost half a million people died from opioid-involved drug overdoses from 1999 to 2018, including nearly 50,000 deaths annually in recent years.^1,2^ Although the crisis is now dominated by overdoses involving illicitly manufactured fentanyl, nearly one-third of opioid overdose deaths involved prescription opioids in 2018.^2,3^

Opioid stewardship measures aiming to limit supply and mitigate harms of prescription opioids in primary care settings have been a major component of the national response to the epidemic.^4–11^ Although these measures, including the 2016 Centers for Disease Control and Prevention (CDC) opioid prescribing guidelines,^12^ have corresponded with substantial reductions in opioid prescribing,^13–17^ the rate of prescribing in the USA remains higher than any other nation and varies widely throughout the country.^13,18,19^ Evidence regarding the effects of opioid prescribing policies is largely limited to opioid prescribing outcomes.^20–26^ However, several studies have linked reduction or discontinuation of prescribed opioids to adverse patient outcomes, including dropping out of care,^27^ illicit use of opioids,^28,29^ and death by overdose and suicide.^30,31^ In light of these risks, critical examination of the effects of specific policies on both opioid prescribing and unintended patient outcomes, such as illicit opioid use, is needed to identify strategies that are both effective and safe.

In 2013–2014, a safety-net primary care clinic in San Francisco, CA, implemented policies guiding opioid prescription refills and the use of urine toxicology testing and established a committee to discuss treatment decisions for complex patient cases. Understanding the effects of these interventions on opioid prescribing and on unintended negative outcomes can inform opioid prescribing policies and practices.

We used a retrospective cohort design to evaluate the combined effects of these two policies and committee on opioid prescribing, use of heroin, and use of opioid analgesics not prescribed to the patient.

## METHODS

### Parent Study

Data were from a retrospective cohort study of 602 publicly insured patients prescribed opioids for chronic non-cancer pain in San Francisco’s safety-net health network.^28^ Eligible participants were ≥18 years old, able to communicate in English, and prescribed opioids for ≥3 months from 2013 to 2015. Participants who were contacted and agreed to participate were seen for a single in-person visit in 2017 or 2018. Additional details regarding the recruitment process are described in the Supplementary Information. Study activities were approved by the University of California San Francisco Institutional Review Board.

### Policies Under Study

The first policy (effective July 1, 2013) outlined procedures for refilling opioid medications when patients requested early or late refills or reported lost or stolen opioid medications. The policy established requirements for increased monitoring and a treatment plan assessment (including possible dose modification) in the case of multiple early refill requests or other concerning behaviors.

The second policy (effective July 1, 2014) required that patients undergo urine toxicology testing prior to initiating opioid therapy and at least annually while continuing therapy. The policy also outlined procedures for when urine toxicology results were inconsistent with prescribed medications (e.g., negative for the prescribed opioid, positive for illicit substances), including a treatment plan assessment and possible opioid discontinuation with a taper.

A new committee began meeting in early 2014 to facilitate multidisciplinary group discussion of patient-centered treatment plans for patients who were prescribed opioids under complex or challenging circumstances (e.g., patients with substance use histories, those prescribed particularly high opioid dosages). The committee was not explicitly tasked with decreasing opioid dose for patients on high doses, but generally provided recommendations to maximize non-opioid treatments and consider dose reduction, especially in high-risk cases.

Because the policies were implemented close together in time, only the combined policy effects are evaluated. Detailed descriptions and documentation for the policies and committee (the interventions) are provided in the Supplementary Information.

### Clinics Under Study

The clinic that implemented the interventions (treated clinic) predominately provided primary care to adults experiencing homelessness, residents of permanent supportive housing, and other members of San Francisco’s downtown neighborhoods, which are characterized by concentrated socioeconomic disadvantage and elevated rates of drug overdose mortality.^32–34^

To evaluate the impacts of the interventions among patients at the treated clinic, we leveraged the experience of patients at a control clinic that is in the same network and serves a demographically similar population as the treated clinic, but that did not implement the interventions. We used patients at a clinic that provided integrated primary and specialty care to HIV-infected patients. The control clinic is unique among the network’s clinics in that, during a time of both national and local reductions in opioid prescribing, it did not implement formal policies to reduce prescribing, whereas other clinics in the network did. In addition, the control clinic is partially managed by a local university and not subject to the same oversight as other clinics in the network.

### Analysis Sample

The analysis was restricted to patients who were prescribed opioids and receiving care from either the treated or control clinic when the first policy was implemented (July 1, 2013). Patients in the analysis sample were required to have started receiving care from the network on January 1, 2012, or earlier; all retrospective study measures were collected starting January 1, 2012, so this restriction ensured that all patients had complete follow-up during the entire period prior to any policy implementation (i.e., January 1, 2012, through June 30, 2013). Measures from pre-policy period were used to assess trends in outcomes during the pre-policy period as well as to generate baseline covariates for estimating propensity scores.

### Study Procedures

Clinical measures were manually abstracted from electronic medical charts for January 1, 2012, through the date of the study visit. Measures included opioid prescriptions (opioid type, dose, quantity per 30 days), emergency department (ED) visits and their opioid-relatedness, and exposure to opioid stewardship activities (controlled substance monitoring program [CSMP] checks, “yellow flag” behaviors documented by the provider [e.g., early refill requests, suspected diversion], and controlled substance agreements).

Self-reported use frequency of heroin, non-prescribed opioid analgesics, cocaine, methamphetamine, and alcohol was collected for each calendar quarter from 2012 until the date of the study visit through an in-person historical reconstruction interview procedure described in the Supplementary Information and elsewhere.^28^ Demographics (e.g., age, education) were also collected at the study visit.

### Baseline Covariates

Baseline covariates included gender, race/ethnicity, age, education, and the following measures corresponding to the full 18-month period prior to the first policy implementation: use of heroin, non-prescribed opioid analgesics, alcohol, methamphetamine, and cocaine; any ED visits; any opioid-related ED visits; any controlled substance agreements; any CSMP checks; any yellow flag behavior indicated by the provider; and mean opioid dose.

### Outcomes

Six study outcomes were assessed for each patient for the 1-year pre-policy period (July 1, 2012–June 30, 2013), implementation period (July 1, 2013–June 30, 2014), and three post-policy periods (July 1, 2014–June 30, 2015; July 1, 2015–June 30, 2016; July 1, 2016–June 30, 2017). We refer to the implementation period and three post-policy periods as the first, second, third, and fourth post-policy periods.

Outcomes included whether the patient had an active opioid prescription on the last day of each period, their mean opioid dose in morphine milligram equivalents (MME; calculated among days the patient was prescribed any opioids),^35,36^ any heroin use and use frequency, and any non-prescribed opioid analgesic use and use frequency. To avoid counting temporary discontinuations of opioid prescriptions, a patient was only considered to not be on opioids at the end of each period if they were not prescribed opioids for a period of at least 3 months including the last day of a period. Use frequency of heroin and non-prescribed opioid analgesics was operationalized as sequential integers ranging from 0 to 6 corresponding to none, once, intermittently, less than weekly or weekly, multiple times per week, and daily or nearly daily. Since these measures were collected quarterly, we used the maximum value within each post-policy period.

All patients were included in the analysis for all outcomes and post-policy periods, except for the mean MME outcome. For this outcome, patients who were not prescribed opioids during a post-policy period were excluded from the analysis for the post-policy period.

### Statistical Analysis

Associations between the interventions and each of the six outcomes were assessed using a conditional difference-in-differences approach, which incorporates propensity score weighting to balance covariates that may be associated with differential outcome dynamics among the treated and control groups.^37^ Additional details are provided in the Supplementary Information.

We used logistic regression to estimate propensity scores using all baseline covariates, which we hypothesized may impact outcome trends, as independent variables. To assess covariate balance achieved via propensity scores, baseline characteristics of treated and control groups were compared using standardized mean differences (SMDs) in the original sample and after weighting each patient by the inverse estimated probability of receiving their observed treatment.^38^

We applied the conditional difference-in-differences weighting estimator to each outcome and post-policy period and calculated 95% bootstrap confidence intervals using the percentile method with 2000 iterations.

We conducted a sensitivity analysis using alternative specifications for the propensity score models, which is described in the Supplementary Information.

## RESULTS

### Sample Characteristics

The analysis included 151 (55%) treated clinic patients and 122 (45%) control clinic patients. Patient characteristics by clinic are presented in Table 1.

**Table 1:** Patient Characteristics by Clinic

Patient characteristics	Entire sample (n=273)		Treated clinic (n=151)		Control clinic (n=122)	
	n	%	n	%	n	%
Age, mean (SD)	51.4	8.5	52.5	8.0	49.9	8.9
Gender						
Male	175	64.1	86	57.0	89	73.0
Female	77	28.2	51	33.8	26	21.3
Transgender or other	21	7.7	14	9.3	7	5.7
Race						
Non-Hispanic white	95	34.8	58	38.4	37	30.3
Non-Hispanic black	99	36.3	56	37.1	43	35.2
Hispanic	38	13.9	13	8.6	25	20.5
Non-Hispanic other/mixed race	41	15.0	24	15.9	17	13.9
Education						
Less than high school	63	23.1	35	23.2	28	23.0
High school graduate	87	31.9	52	34.4	35	28.7
Some college, associate’s degree, or vocational training	94	34.4	49	32.5	45	36.9
Bachelor’s degree or higher	29	10.6	15	9.9	14	11.5
HIV positive	141	51.6	24	15.9	117	95.9
Mean pre-policy opioid dose (MME), mean (SD)	269.9	549.0	264.8	490.9	276	615.5
Any pre-policy heroin use	29	10.6	18	11.9	11	9.0
Any pre-policy non-prescribed opioid analgesic use	40	14.7	25	16.6	15	12.3
Any pre-policy alcohol use	155	56.8	83	55.0	72	59.0
Any pre-policy cocaine use	73	26.7	38	25.2	35	28.7
Any pre-policy methamphetamine use	47	17.2	29	19.2	18	14.8
Any pre-policy emergency department visits	103	37.7	60	39.7	43	35.2
Any pre-policy opioid-related emergency department visits	34	12.5	17	11.3	17	13.9
Any pre-policy controlled substance agreements	40	14.7	30	19.9	10	8.2
Any pre-policy yellow flag behaviors	80	29.3	49	32.5	31	25.4
Any pre-policy CSMP checks	10	3.7	9	6.0	1	0.8

### Covariate Balance

There were substantial reductions in the SMDs of baseline covariates between treated and control groups after weighting by the inverse probability of treatment received (Supplemental Table 1). However, 3 out of 20 covariates had SMDs > 10 after weighting: having a bachelor’s degree or higher (12.0), any ED visit (−10.8), and any opioid-related ED visit (−10.7).

### Conditional Difference-in-Differences Estimates

**Figure 1:**
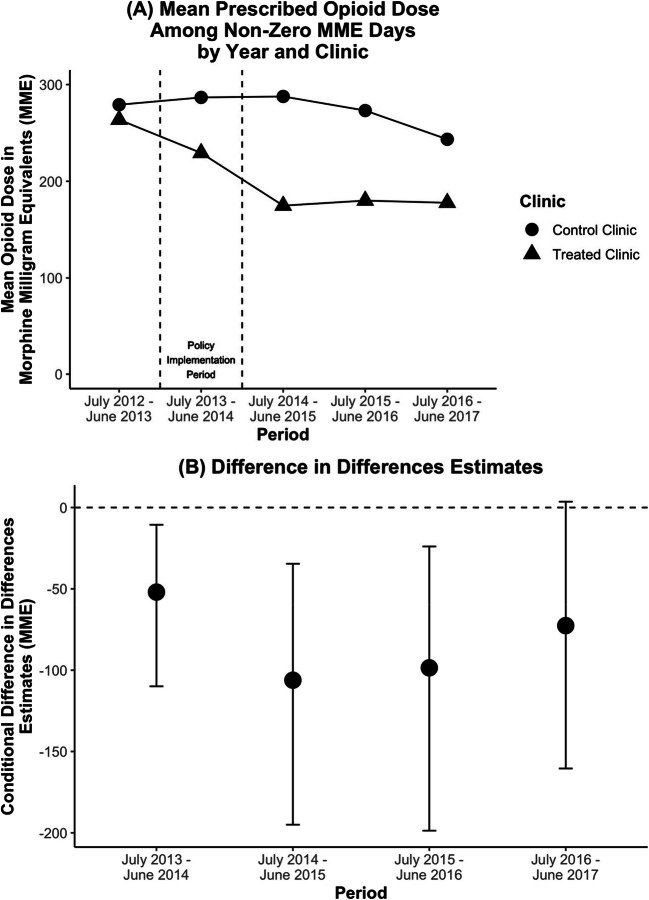
**A Outcome trends by clinic and B conditional difference-in-differences estimates for the mean opioid dose outcome.**

The interventions were associated with a reduction in mean opioid dose in the first three post-policy years (year 1 conditional difference-in-differences estimate: −52.0 MME [95% confidence interval: −109.9, −10.6]; year 2: −106.2 MME [−195.0, −34.6]; year 3: −98.6 MME [−198.7, −23.9]; year 4: −72.6 MME [−160.4, 3.6]) (Table 2 and Fig. 1). The analysis for this outcome included 273, 260, 248, and 239 patients who were prescribed opioids for at least 1 day during post-policy periods one to four, respectively.

**Table 2:** Conditional Difference-in-Differences Estimates for the Association Between Opioid Prescribing Interventions and Patient Outcomes

Patient outcome	Year 1		Year 2		Year 3		Year 4	
	Est.	95% CI	Est.	95% CI	Est.	95% CI	Est.	95% CI
Opioid prescription (absolute %)	3.5	−8.4, 22.5	0.7	−11.7, 22.0	6.6	−6.3, 27.2	2	−12.3, 24.5
Mean opioid dose (MME)	−52.0	−109.9, −10.6	−106.2	−195.0, −34.6	−98.6	−198.7, −23.9	−72.6	−160.4, 3.6
Heroin use								
Any use (absolute %)	1.2	−3.7, 6.6	0.9	−4.6, 7.1	−0.4	−8.9, 8.8	2.3	−4.2, 11.6
Frequency of use (ordinal use scale)	0.06	−0.10, 0.23	0.03	−0.16, 0.23	0.06	−0.24, 0.53	0.1	−0.19, 0.48
Non-prescribed opioid analgesic use								
Any use (absolute %)	1.3	−1.3, 4.0	3.0	−1.9, 9.6	5.2	−0.1, 11.2	4.6	−5.0, 13.1
Frequency of use (ordinal use scale)	0.03	−0.07, 0.13	0.14	−0.09, 0.41	0.21	0.00, 0.47	0.12	−0.16, 0.41

*Est*., estimate

There was no evidence that the combined interventions were associated with whether patients were prescribed opioids at the end of each period (Supplemental Figure 2) or heroin use or use frequency (Supplemental Figures 3–4).

Although confidence intervals included the null, estimates suggested a possible positive association between the combined interventions and non-prescribed opioid analgesic use (year 3: 5.2 absolute percentage points [−0.1, 11.2]) and use frequency (year 3: 0.21 ordinal frequency scale points [0.00, 0.47]) in the third post-policy year (Supplemental Figures 5–6).

Sensitivity analysis results were largely consistent with the main results and are presented in Supplemental Table 2.

## DISCUSSION

We found that the interventions were associated with reductions in opioid dose in the first 3 years and possibly with increases in non-prescribed opioid analgesic use. There was no evidence that the interventions were associated with changes in the overall proportion of patients prescribed opioids or in heroin use.

The interventions were associated with over a 100 MME reduction (40% relative change from the pre-intervention dose), which brought the mean opioid dose among treated clinic patients below 200 MME, a dose deemed high-risk and targeted for reduction by policies at the Veterans Administration Health System.^39^ The interventions did not have the explicit aim of reducing patients’ opioid dose but rather aimed to mitigate concerning patient behavior and improve safety. Our findings suggest that policies guided by this strategy may provide an effective approach for reducing opioid dose among patients receiving opioid therapy.

The dose reductions were greatest in the second and third post-policy years but were largely attenuated by the fourth year. The declining trend in opioid dose among control clinic patients suggests that secular trends in opioid prescribing may have caught up to reductions achieved by the interventions. Although the control clinic did not implement specific opioid prescribing policies, our study period overlaps with several trends and policies that likely affected prescribing trends at both the treated and control clinics. There were national reductions in prescribed opioid dose during this time,^16,17^ which were likely driven by an increasing awareness of the risks associated with prescription opioids^40–44^ and further influenced by the release of the CDC’s prescribing guidelines in 2016.^14^ There were also several state and local changes that aimed to promote safer opioid prescribing during this period (details provided in the Supplementary Information). Our results suggest that these changes, among others, were sufficient to gradually reduce the prescribed opioid dose without the explicit interventions implemented at the treated clinic, though not with the rapidity achieved by the interventions.

There was no association between the interventions and the proportion of patients prescribed opioids. However, to participate in the parent study, patients were required to complete an in-person visit in either 2017 or 2018, which may have systematically excluded patients who had dropped out of care, died, or been otherwise difficult to locate since the interventions were implemented in 2013–2014. Opioid discontinuation and dose reductions have been linked to both dropping out of care^27^ and mortality,^30,31^ suggesting the possibility that patients who had their opioids discontinued may be under-represented in our sample. An unpublished quality improvement analysis conducted at the treated clinic found a 52% reduction in the number of patients on long-term opioid therapy from 2012 to 2015, which is substantially larger than the 15% reduction observed among our treated clinic sample over a similar time frame.^45^ This difference suggests that patients who had their opioids discontinued are under-represented in our treated clinic sample; unfortunately, we have no way to make a similar assessment for our control clinic sample. However, under the assumption that undersampling of opioid-discontinued patients occurred at the same rate across the treated and control clinics, our estimate of a true effect would be biased towards the null. Thus, our null effect estimate for this measure should be interpreted with caution. To mitigate the threat of such biases, it is important that future studies evaluate opioid prescribing policies using entire populations of interest or truly representative samples.

The results were suggestive of a positive association between the interventions and use of non-prescribed opioid analgesics, although confidence intervals included the null. It is notable that use of non-prescribed opioid analgesics increased among both treated and control clinics. From 2015 to 2019, national estimates of past year misuse of pain relievers declined.^46,47^ However, to our knowledge, no studies have assessed trends in non-prescribed opioid analgesic use among patients prescribed opioids during this period; thus, it is unclear whether the trends observed in our sample are generalizable to other populations or unique to the clinics under study. However, multiple studies have linked prescription opioid discontinuation with adverse patient outcomes, including mortality and heroin use.^28–31^ A study among the entire parent study sample observed associations between opioid discontinuations and dose reductions and non-prescribed opioid analgesic use,^28^ which is consistent with our findings that the interventions under study may have increased the use of non-prescribed opioid analgesics. Also, the treated clinic implemented an integrative pain management program to advance the use of multimodal pain treatments in 2016, which was associated with self-reported improvements in pain, social satisfaction, and mental health outcomes among participating patients.^48^ It is plausible that this program and its benefits could have attenuated any adverse impacts of the interventions on non-prescribed opioid use. Regardless, our findings highlight the importance of carefully weighing the potential benefits and harms associated with reducing or discontinuing opioid therapy for individual patients.^49^

The present study has several limitations. First, our convenience sample may not be representative of the entire patient population among the two clinics over the course of the study period. Importantly, our study design required patients to complete an in-person interview at the end of the retrospective study period, which may have systematically exclude patients who dropped out of care or died. Second, our measures of illicit opioid use were obtained via retrospective self-report and thus vulnerable to reporting biases. Third, the patients under study are socioeconomically disadvantaged and have higher rates of substance use and HIV relative to the general population and thus the estimated impacts of these policies may not be generalizable to other patient populations. Fourth, the validity of the conditional difference-in-differences estimator depends on the assumption that any differences in outcome trends not attributable to the treatment are attributable to baseline characteristics that we observed and were able to balance across treated and control clinics using propensity scores, which is not directly testable. Related to this, our propensity score approach did not balance the prevalence of having a bachelor’s degree or higher, or experiencing an ED visit or opioid-related ED visit during the pre-policy period. However, after weighting, the treated clinic patients exhibited a higher prevalence of having a bachelor’s degree or higher, and lower baseline prevalences of ED visits and opioid-related ED visits relative to control clinic patients. If these covariates were correlated with post-intervention outcome trends, this imbalance would suggest a lower-risk treated population and we anticipate a bias towards the null. Specifically, the weighted treated sample has a risk profile that makes them less likely, relative to the weighted control sample, to experience a dose reduction or to increase use of non-prescribed opioid analgesics in the absence of the interventions. Lastly, our analytic approach and choice of control group assume that outcome trends are independent of HIV status, conditional on other observed patient characteristics. Although levels of outcomes may differ by HIV status, we have no reason to expect trends in outcomes to differ after accounting for other demographic and clinical characteristics.

Despite these limitations, the present study offers a rigorous evaluation of the effects of specific opioid prescribing policies on opioid prescribing and illicit opioid use. Among this sample, we found that policies addressing concerning behavior, such as multiple early refill requests or abnormal urine toxicology results, and a patient-centered committee designed to facilitate challenging treatment decisions were associated with a substantial reduction in mean opioid dose and possibly with an increase in non-prescribed opioid analgesic use. Such empirical evidence is essential for informing the path forward as we seek a balance between reducing opioid prescribing and minimizing harms for affected patients.

## Supplementary Information

ESM 1 (DOCX 1861 kb)

ESM 2 (PDF 690 kb)

ESM 3 (PDF 1072 kb)

ESM 4 (PDF 197 kb)
