# Supplementary material for: Evaluating the Effects of Opioid Prescribing Policies on Patient Outcomes in a Safety-net Primary Care Clinic
Source: J Gen Intern Med. 2021 Jun 25;37(1):117–24. doi: 10.1007/s11606-021-06920-4 (PMC8738839; doi:10.1007/s11606-021-06920-4)
Supplement: Supplementary file 1 — (DOCX 1861 kb) [file 11606_2021_6920_MOESM1_ESM.docx]

**APPENDIX A – SUPPLEMENTAL METHODS AND RESULTS**

***Recruitment Process***

To be eligible for the parent study, patients needed to be ≥18 years of age, prescribed opioid pain relievers exclusively for chronic non-cancer pain for ≥3 months from 2013–2015, and able to communicate in English. Potential participants were identified through patient registries maintained by each San Francisco Health Network clinic. Providers were contacted to obtain permission to contact patients in 2017-2018. Finally, participants were contacted and agreed to participate were seen for a single in-person visit in 2017 or 2018, which included an interview to collect retrospective substance use patterns (including heroin and non-prescribed opioid analgesics) and other demographic and behavioral information.

Although the parent study included patients from several clinics throughout the San Francisco Health Network, this analysis only included patients from two clinics. In total, 1,067 potentially eligible patients were identified from patient registries associated with the two clinics included in this analysis. Provider permission to contact was obtained for 780 patients. Patients for which permission was not obtained included 119 for whom response was not received or patient was deemed ineligible and 168 for which permission was declined. A total of 565 patients were contacted regarding the parent study. Patients who were not contacted included 39 from whom contact was not yet attempted and 176 who could not be reached. Among the 565 patients contacted, 424 agreed to participate, 118 declined, and 23 were deemed ineligible. Among the 424 patients who agreed to participate, 370 were successfully enrolled and completed the study visit. Among these 370 patients, the final sample included 273 patients who were prescribed opioids and receiving care from either the treated or control clinic when the first policy was implemented (July 1, 2013) and started receiving care from the San Francisco Health Network on January 1, 2012 or earlier.

***Historical Reconstruction Interview Procedure***

Self-reported use frequency of heroin, non-prescribed opioid analgesics, cocaine, methamphetamine, and alcohol were collected for each calendar quarter from 2012 until the date of the study visit using an in-person historical reconstruction interview procedure, which has been described in detail elsewhere.^1^

The historical reconstruction interview involved generating and displaying a personal timeline from January 1, 2012 to the interview date (2017 or 2018) using significant autobiographical (e.g., marriage, health events, housing transitions) and societal events (e.g., local news or events, natural disasters, elections) specific to each participant. Measures that incorporate the use of autobiographical landmarks during collection have been shown to be valid and reliable in assessing alcohol consumption.^2,3^ Moreover, the use of the visual timeline provided cues to facilitate more accurate recall of historical information.^4^ This visually displayed personal timeline served as a foundation for the reconstruction and dating of retrospective substance use patterns.

In order to obtain retrospective substance use patterns, we adapted a structured interview procedure from the Lifetime Drinking History interview and the Lifetime Drug Use Questionnaire,^5,6^ which both construct substance use patterns over an entire lifetime in by defining distinct periods of use. These periods of use are characterized by quantity of frequency of use and are defined by the interviewee. These interview methods have been shown to have strong validity and reliability in retrospective assessment of substance use among multiple populations, including strong correlations with prospective measures,^7^ highly significant test-retest correlations,^2,3,5,8^ and agreement between subject-collateral reports,^9^ for up to 13 years in the past.^7,10^ In our modified approach, we limited the recall period to 2012 onward and mapped use periods to calendar quarters (i.e., January-March, etc.).

Interviews were conducted by trained staff after they had shadowed at least five interviews and been shadowed for an additional three interviews. Sessions were also audio-recorded for review to ensure that data collection was comprehensive and consistent.

***Policies Under Study***

The interventions were established at the treated clinic within a one-year period from July 1, 2013 to July 1, 2014. The purpose of the two policies was to define uniform procedures related to opioid prescribing so as to “clarify expectations and improve safety for patients and staff, reduce risks and harms from opioids, and reduce staff anxiety.” The first policy addressed opioid refills and the second policy addressed the use of urine toxicology testing. The new committee was called the “yellow flag” committee in reference to yellow flag behaviors, which are potentially concerning behaviors in the context of opioid prescribing (e.g., repeated requests for dose escalations, presenting to clinic while intoxicated). The purpose of the yellow flag committee, which began meeting in early 2014, was to establish a formal system for multidisciplinary group discussion of treatment plans for patients who were prescribed opioids under complex or challenging circumstances (e.g., patients with substance use histories, those prescribed particularly high opioid dosages, or those with a history of yellow flag behaviors).

Opioid Refill Policy: Effective July 1, 2013, the opioid refill policy established uniform procedures for refilling opioid medications. Specifically, the policy outlined procedures for refilling opioid medications when patients request early or late refills or report lost or stolen opioid medications or prescriptions. The policy established a limit of one early or late refill in a 12 month period and outlined requirements for increased monitoring and a detailed treatment plan assessment (including possible dose modification) in the case of multiple early or late refill requests or for patients who have exhibited yellow flag behavior. The full policy document from 2013 is provided in Appendix B.

Urine Toxicology Policy: Effective July 1, 2014, the urine toxicology policy established uniform procedures for requesting urine samples from patients for toxicological testing, interpreting results, and taking action based on results. The policy required that all patients prescribed chronic opioids be asked to submit a urine sample for toxicology testing prior to initiating therapy and at least once per 12 months while continuing therapy. More frequent testing was performed as clinically indicated. The policy outlined procedures for when urine toxicology results were inconsistent with prescribing (e.g., negative for the prescribed opioid, or positive for cocaine, methamphetamine, or non-prescribed controlled substances), including a detailed assessment of the patient’s treatment plan and possible opioid discontinuation with a taper. The full policy document from 2014 is provided in Appendix C.

Yellow Flag Committee: The “yellow flag” committee, which was conceived in late 2013 and officially began meeting in early 2014, was designed to discuss treatment plans for the following types of patients: those on opioids whose use has become problematic for the patient, provider, or staff; those with three or more “yellow flag” behaviors; those with a behavioral agreement for disruptive or violent behavior related to opioids; and those on opioids who are at higher risk for overdose or death (e.g., patients with overdose in the past 12 months, patients on high dose opioids [>400 MME per day or >100mg methadone per day for pain]). The committee did not review every case falling into the above categories, which was not feasible due to volume, but sought to identify and review the most complex patient cases involving opioids, which typically fell into one of these categories. Any staff member at the clinic could also request a case review by the committee as well, regardless of reason. Committee composition included a provider (always a medical doctor, but sometimes also a nurse practitioner or physician assistant, who was not the primary care provider), nursing staff and behavioral health staff. The committee included individuals not previously involved in care of the patient to incorporate a new and potentially more objective lens under which the case was reviewed. The committee was not explicitly tasked with decreasing the opioid dose for patients on high doses, but generally did provide recommendations to maximize non-opioid treatments and consider decreasing doses, especially in high risk cases. Discussions included comprehensive understanding of the medical, psychiatric, substance use and psychosocial complexities of the case. The multidisciplinary composition of the group and individualized discussion of cases created a patient-centered approach to the most complex and challenging cases. The committee referral form from 2013 is provided in Appendix D.

We note that the policy documents provided are historical documents and do not necessarily reflect current policies or practices at the clinic under study.

***Pre-Policy Outcome Trends***

The key assumption underlying the standard difference in differences approach in our context is that the change in outcome from pre- to post-policy periods in the control clinic represents the change that would have occurred in the treated clinic had the policies under study not been implemented (i.e., the secular trend). If this “parallel trends” assumption holds, the difference in differences estimate can be interpreted as an unbiased estimate of the effect of the policies on the outcome under study. Although this assumption is fundamentally untestable, its plausibility can be assessed by examining treated and control clinic outcome trends during the pre-policy period. Although not guaranteed, parallel outcome trends during the pre-policy period may suggest that the trends would have remained parallel through the post-policy period had the policies not been implemented. Thus, we plotted the mean outcomes over time during the pre-policy period as well as separate linear regression fits using the individual-level outcomes for each clinic. Because there are only 18 months in the pre-period, we plotted outcomes and fit the regression lines using calendar quarter as the timescale. As a result, the unit of time utilized in assessing pre-policy outcome trends is smaller than that used in the main analysis (i.e., years), which may result in greater outcome variability in the pre-policy assessment.

Pre-policy outcome trends are presented in Supplemental Figure 1. We did not assess pre-policy trends for the binary opioid prescription outcome because the analysis sample was restricted to patients who had an active prescription at the end of the pre-policy period.

***Conditional Difference-in-Differences Estimator***

The associations between the new policies and each of the six outcomes were assessed using a conditional difference-in-differences approach.^11^ The key assumption underlying the standard difference-in-differences approach is that the change in outcome from pre- to post-policy periods in the control clinic represents the change that would have occurred in the treated clinic had the policies under study not been implemented (i.e., the secular trend). However, because visual inspection of outcome trends during the pre-policy period suggested possible violations of this “parallel trends” assumption, we applied a generalization of the difference-in-differences design that relies on the weaker “conditional parallel trends” assumption. The conditional parallel trends assumption posits that any differences in outcome trends in the absence of treatment are the result of differences in observed baseline covariates between the treated and the control group. This approach uses propensity score weighting to balance covariates that may be associated with differential outcome dynamics among the treated and control groups.

As presented elsewhere,^11^ the conditional difference-in-differences estimates for each post-policy period are calculated as follows:


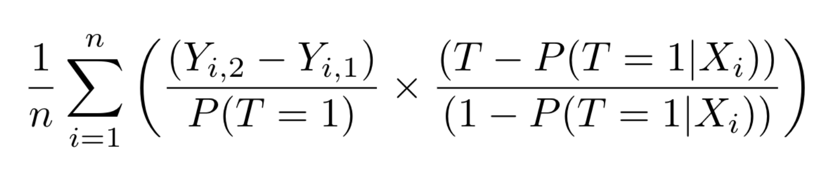


Where *Y_i_*_,2_ is the outcome for patient *i* in period 2 (i.e., a post-policy period) and *Y_i_*_,2_ is the outcome for patient *i* in period 1 (i.e., the pre-policy period). *T_i_* is the treatment indicator for patient *i*, which is equal to 1 if the patient was a patient of the treated clinic and 0 otherwise. *P*(*T*=1|*X_i_*) is the estimated propensity score and *P*(*T*=1) is the empirical proportion of treated clinic patients among the entire sample.

Post-policy outcomes and conditional difference-in-differences estimates for having an opioid prescription at the end of each period, heroin use and use frequency, and non-prescribed opioid analgesic use and use frequency are presented in Supplemental Figures 2-6.

***Sensitivity Analysis***

Because matching on pre-period outcome levels can bias difference-in-difference estimates when pre-period outcome levels are correlated with treatment assignment,^12^ we conducted a sensitivity analysis in which we excluded the relevant pre-period outcome as an independent variable in the propensity score models. Specifically, we excluded mean opioid dose when estimating the propensity scores for the opioid prescription outcomes, heroin use for the heroin outcomes, and non-prescribed opioid analgesic use for the non-prescribed opioid analgesic outcomes.

The results of this sensitivity analysis were largely consistent with the main results and are presented in Supplemental Table 2.

***State and Local Policies Effecting Opioid Prescribing During the Study Period***

Although the control clinic did not implement specific opioid prescribing policies, our study period overlaps with number of state and local opioid-related changes that likely to contributed to prescribing trends at both the treated and control clinics. First, the Medical Board of California adopted its own prescribing guidelines in 2014,^13^ which were similar to the CDC’s 2016 opioid prescribing guidelines and may have led to reductions in opioid prescribing across California. Second, the network that includes the clinics under study formed a pain management workgroup in 2012 to promote responsible prescribing across all the network’s clinics. Third, the managed care organization that oversees the management of the majority of Medicaid clients in San Francisco implemented dose and quantity limits on opioid prescribing in 2014.

***References***

1. Coffin PO, Rowe C, Oman N, et al. Illicit opioid use following changes in opioids prescribed for chronic non-cancer pain. Fischer G, ed. *PLoS ONE*. 2020;15(5):e0232538. doi:10.1371/journal.pone.0232538

2. Chaikelson JS, Arbuckle TY, Lapidus S, Gold DP. Measurement of lifetime alcohol consumption. *J Stud Alcohol*. 1994;55(2):133-140.

3. Sobell LC, Sobell MB, Riley DM, et al. The reliability of alcohol abusers’ self-reports of drinking and life events that occurred in the distant past. *J Stud Alcohol*. 1988;49(3):225-232.

4. Means B, Nigam A, M Z, EF L, MS D. *Autobiographical Memory for Health-Related Events*. National Center for Health Statistics; 1989.

5. Czermak C, Lehofer M, Gasser-Steiner P, et al. Test-retest reliability of a lifetime drug use questionnaire. *Addict Behav*. 2005;30(2):361-368. doi:10.1016/j.addbeh.2004.05.005

6. Skinner HA, Sheu WJ. Reliability of alcohol use indices. The Lifetime Drinking History and the MAST. *J Stud Alcohol*. 1982;43(11):1157-1170.

7. Koenig LB, Jacob T, Haber JR. Validity of the lifetime drinking history: a comparison of retrospective and prospective quantity-frequency measures. *J Stud Alcohol Drugs*. 2009;70(2):296-303. doi:10.15288/jsad.2009.70.296

8. Jacob T, Seilhamer RA, Bargeil K, Howell DN. Reliability of Lifetime Drinking History among alcohol dependent men. *Psychol Addict Behav*. 2006;20(3):333-337. doi:10.1037/0893-164X.20.3.333

9. Gladsjo JA, Tucker JA, Hawkins JL, Vuchinich RE. Adequacy of recall of drinking patterns and event occurrences associated with natural recovery from alcohol problems. *Addict Behav*. 1992;17(4):347-358.

10. Vlahov D, Munoz A, Anthony JC, Cohn S, Celentano DD, Nelson KE. Association of drug injection patterns with antibody to human immunodeficiency virus type 1 among intravenous drug users in Baltimore, Maryland. *Am J Epidemiol*. 1990;132(5):847-856. doi:10.1093/oxfordjournals.aje.a115727

11. Abadie A. Semiparametric Difference-in-Differences Estimators. *Review of Economic Studies*. 2005;72:1-19.

12. Daw JR, Hatfield LA. Matching and Regression to the Mean in Difference-in-Differences Analysis. *Health Serv Res*. 2018;53(6):4138-4156. doi:10.1111/1475-6773.12993

13. Medical Board of California. *Guidelines For Prescribing Controlled Substances For Pain*.; 2014.

| **Supplemental Table 1:** Standardized mean differences for baseline characteristics between treated and control clinic patients in original and weighted samples | | |
| --- | --- | --- |
|  | **Standardized Mean Difference** | |
| **Baseline Characteristics** | **Original Sample** | **Weighted Sample** |
| Age, *mean (SD)* | 30.7 | -6.7 |
| **Gender** |  |  |
| Proportion female | 28.1 | -2.69 |
| Proportion transgender or other | 13.4 | -2.59 |
| **Race** |  |  |
| Proportion non-Hispanic black | 3.82 | -3.8 |
| Proportion Hispanic | -34.1 | -5.51 |
| Proportion non-Hispanic other/mixed race | 5.48 | -1.1 |
| **Education** |  |  |
| Proportion high school graduate | 12.3 | -3.29 |
| Proportion some college, associate's degree, or vocational training | -9.29 | -5.7 |
| Proportion bachelor's degree or higher | -4.97 | 12.0 |
| **Mean pre-policy opioid dose (MME), *mean (SD)*** | -2.03 | -0.612 |
| **Any pre-policy heroin use** | 9.46 | 6.23 |
| **Any pre-policy non-prescribed opioid analgesic use** | 12.1 | -4.67 |
| **Any pre-policy alcohol use** | -8.16 | -4.08 |
| **Any pre-policy cocaine use** | -7.92 | -0.804 |
| **Any pre-policy methamphetamine use** | 11.8 | 1.71 |
| **Any pre-policy emergency department visits** | 9.25 | -10.8 |
| **Any pre-policy opioid-related emergency department visits** | -8.04 | -10.7 |
| **Any pre-policy controlled substance agreements** | 34 | -4.86 |
| **Any pre-policy yellow flag behaviors** | 15.5 | -3.02 |
| **Any pre-policy CSMP checks** | 28.6 | -6.81 |

| **Supplemental Table 2:** Conditional difference in difference estimates for the association between opioid prescribing interventions and patient outcomes (pre-period outcome levels excluded from propensity score estimation) | | | | | | | | |
| --- | --- | --- | --- | --- | --- | --- | --- | --- |
|  | **Year 1** | | **Year 2** | | **Year 3** | | **Year 4** | |
| **Patient Outcome** | ***Est.*** | **(95% CI)** | ***Est.*** | **(95% CI)** | ***Est.*** | **(95% CI)** | ***Est.*** | **(95% CI)** |
| **Opioid prescription**  **(absolute %)** | 2.4 | (-8.9, 20.0) | -0.5 | (-12.3, 19.6) | 5.3 | (-7.0, 24.0) | 1.3 | (-12.5, 22.5) |
| **Mean opioid dose**  **(MME)** | -48.8 | (-109.0, -5.0) | -102.3 | (-191.6, -28.7) | -90.0 | (-189.0, -5.6) | -58.7 | (-156.7, 38.6) |
| **Heroin use** |  |  |  |  |  |  |  |  |
| Any use  (absolute %) | 0.7 | (-4.5, 5.0) | 0.4 | (-5.3, 5.7) | -1.5 | (-10.0, 6.1) | 1.4 | (-4.9, 8.1) |
| Frequency of use  (ordinal use scale) | 0.05 | (-0.12, 0.20) | 0.01 | (-0.18, 0.20) | 0.01 | (-0.27, 0.31) | 0.06 | (-0.24, 0.34) |
| **Non-prescribed**  **opioid analgesic use** |  |  |  |  |  |  |  |  |
| Any use  (Absolute %) | 1.3 | (-1.3, 4.0) | 2.8 | (-2.1, 8.5) | 5 | (-0.4, 10.8) | 4.4 | (-5.1, 12.8) |
| Frequency of use  (ordinal use scale) | 0.03 | (-0.07, 0.13) | 0.13 | (-0.10, 0.37) | 0.2 | (-0.01, 0.45) | 0.11 | (-0.16, 0.39) |
| Est. = estimate |  |  |  |  |  |  |  |  |

**Supplemental Figure 1: Pre-policy outcome trends for (A) mean opioid dose (B) any heroin use, (C) heroin use frequency, (D) any non-prescribed opioid analgesic use, and (E) non-prescribed opioid analgesic use frequency**


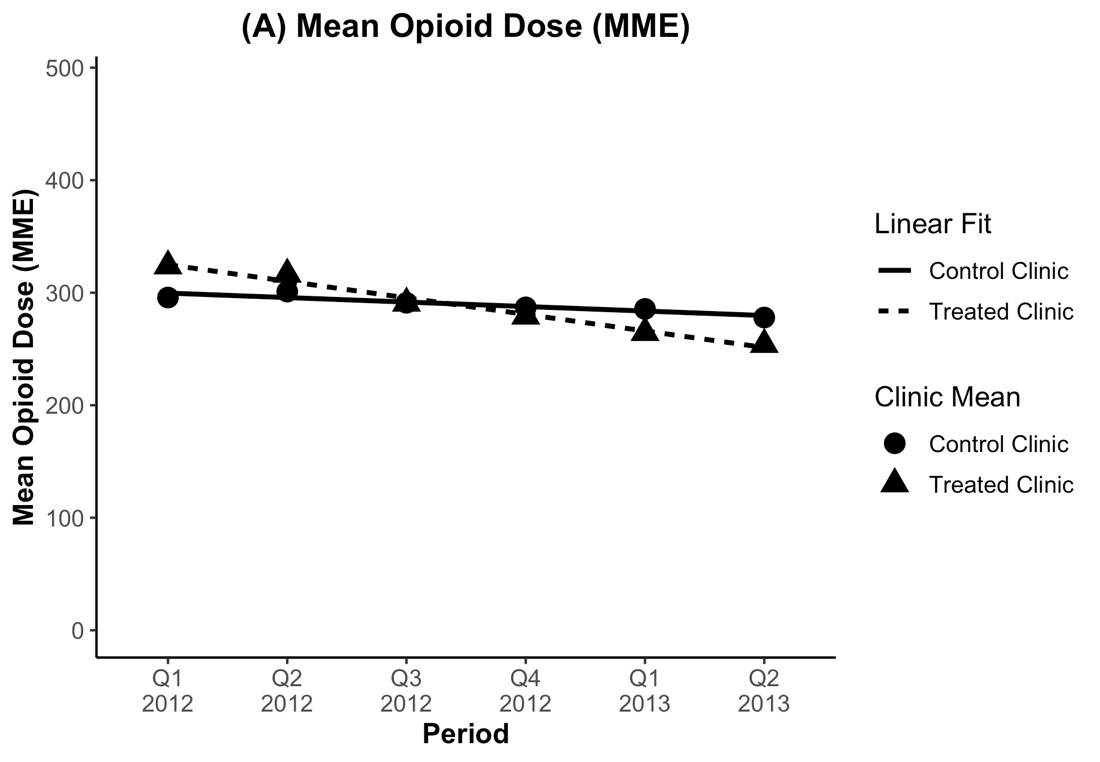


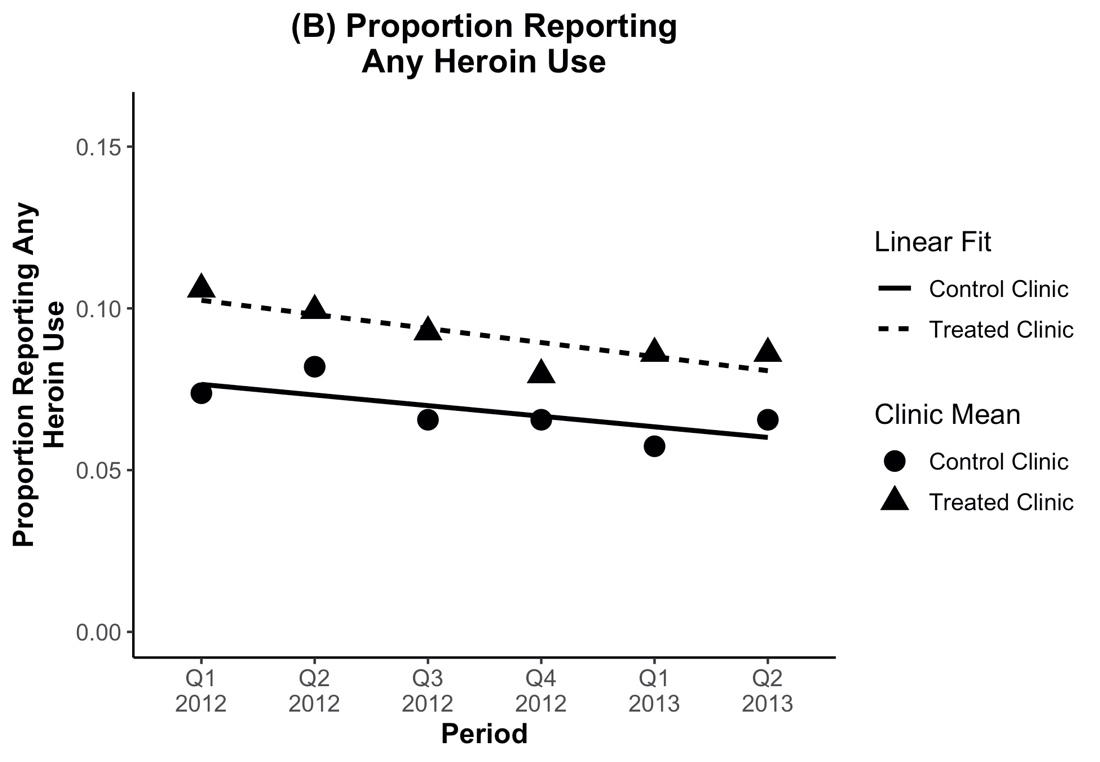


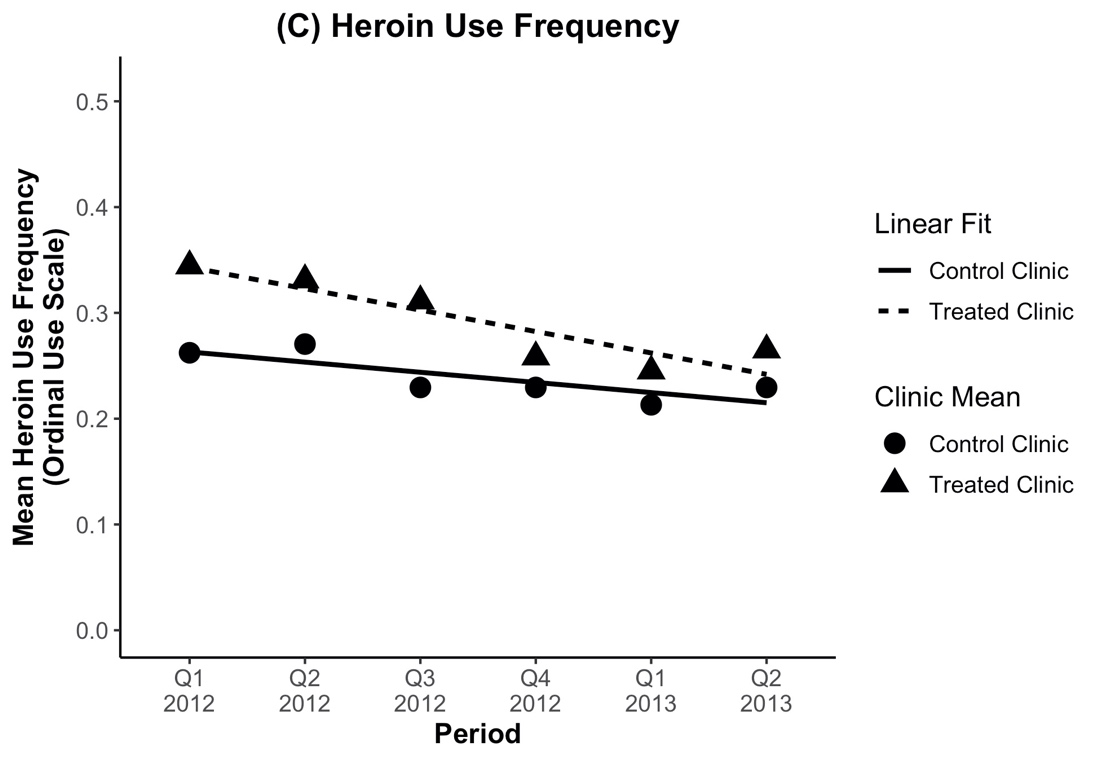


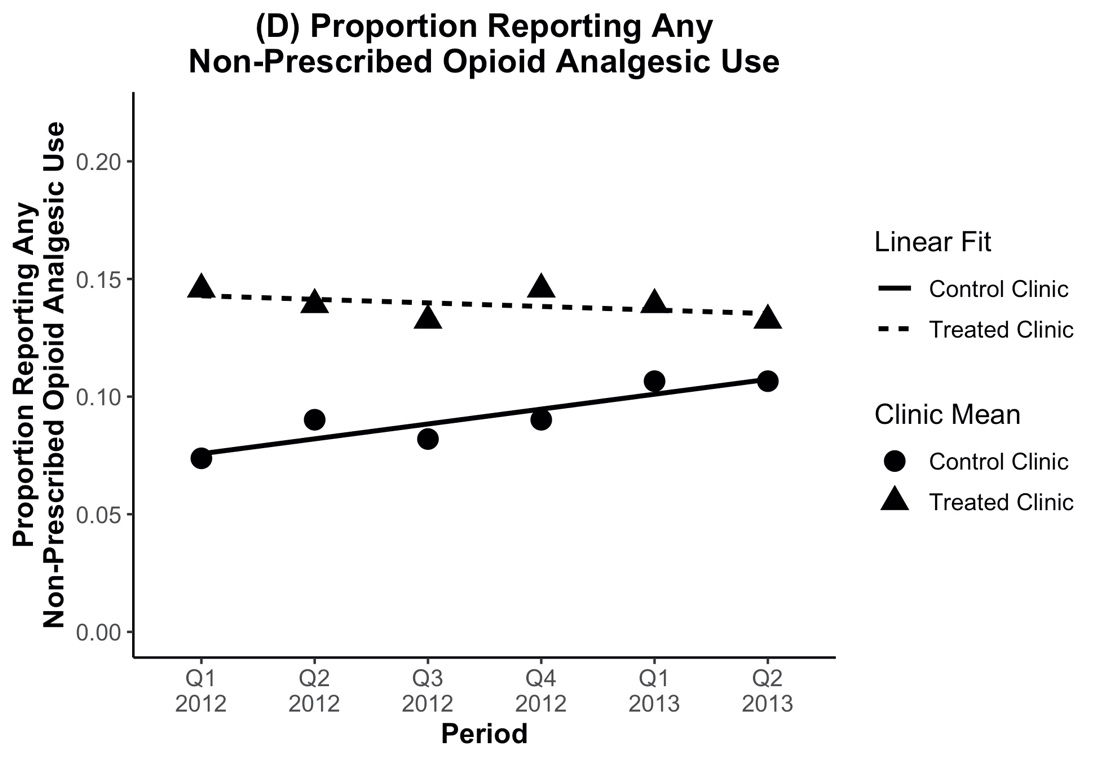


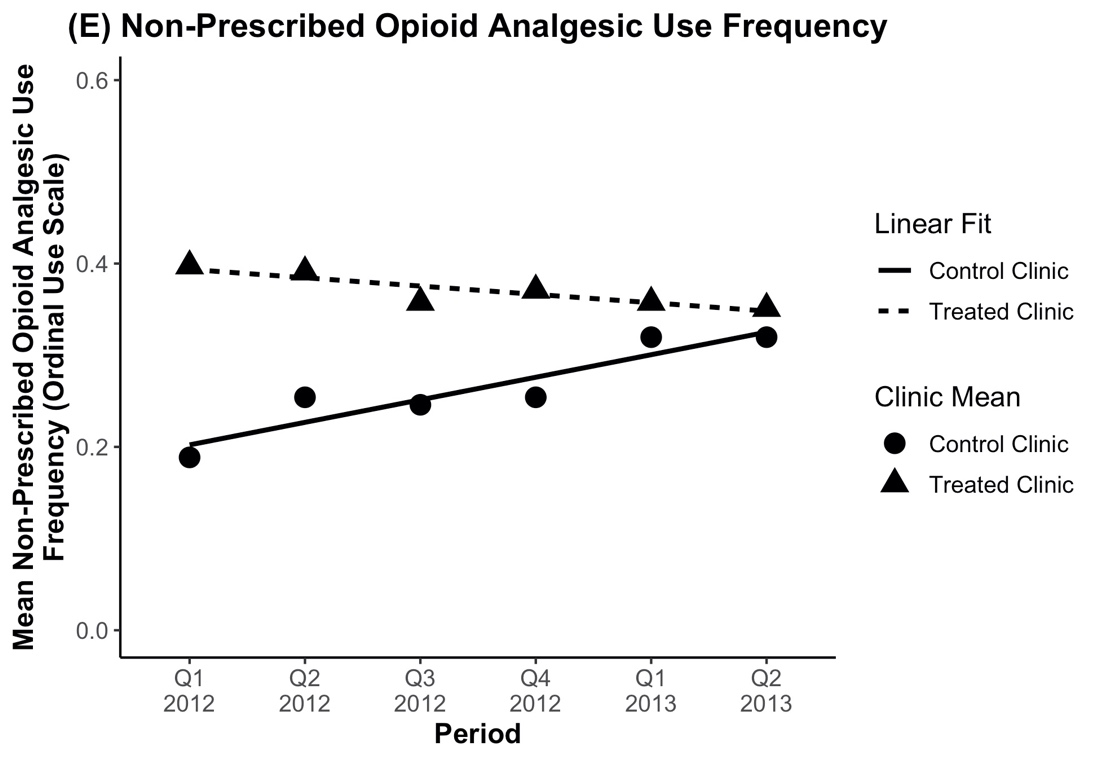


**Supplemental Figure 2: (A) Outcome trends by clinic and (B) conditional difference in differences estimates for the binary opioid prescription outcome**

**
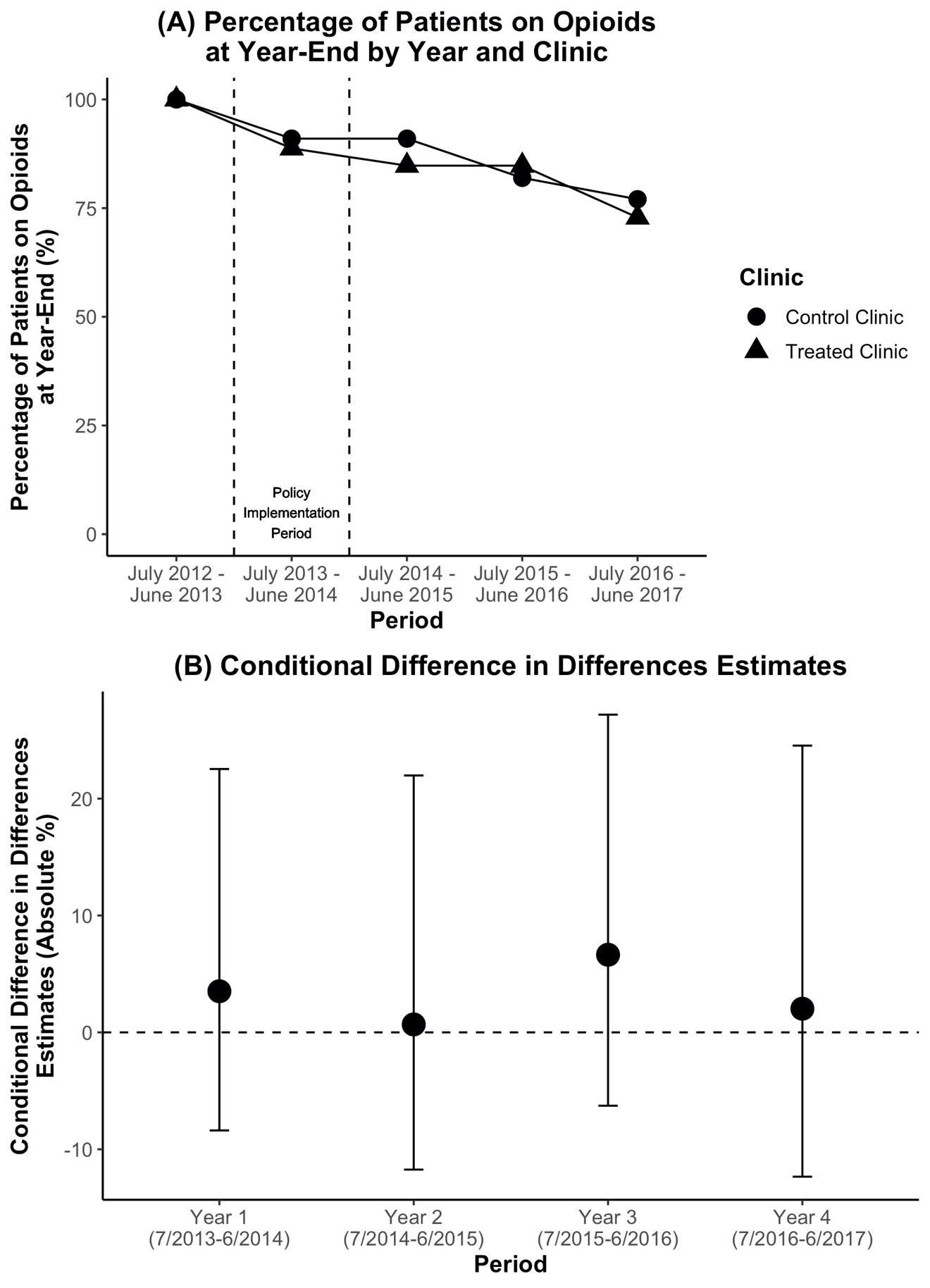
**

**Supplemental Figure 3: (A) Outcome trends by clinic and (B) conditional difference in differences estimates for the any heroin use outcome**

**
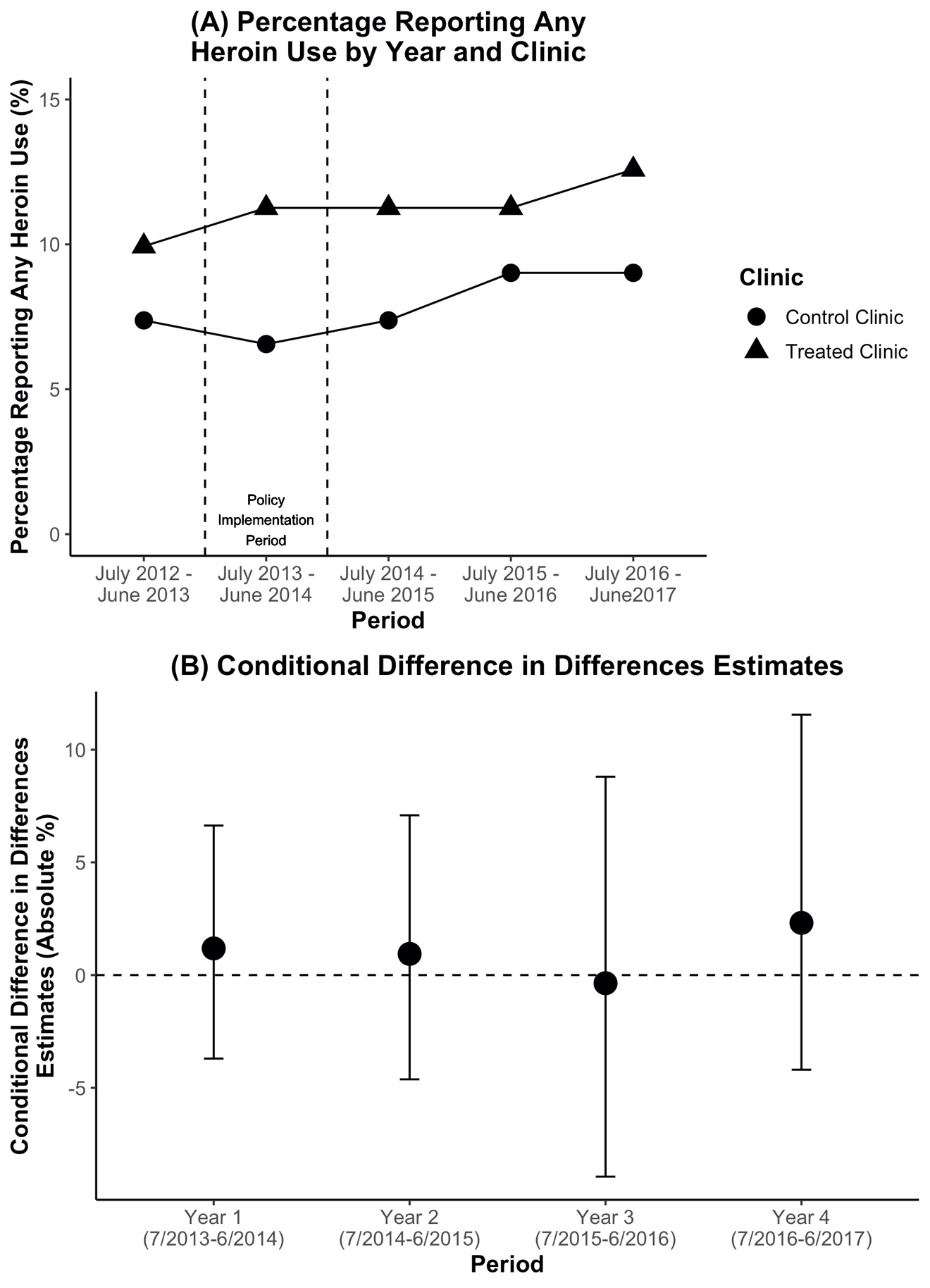
**

**Supplemental Figure 4: (A) Outcome trends by clinic and (B) conditional difference in differences estimates for heroin use frequency outcome**

**
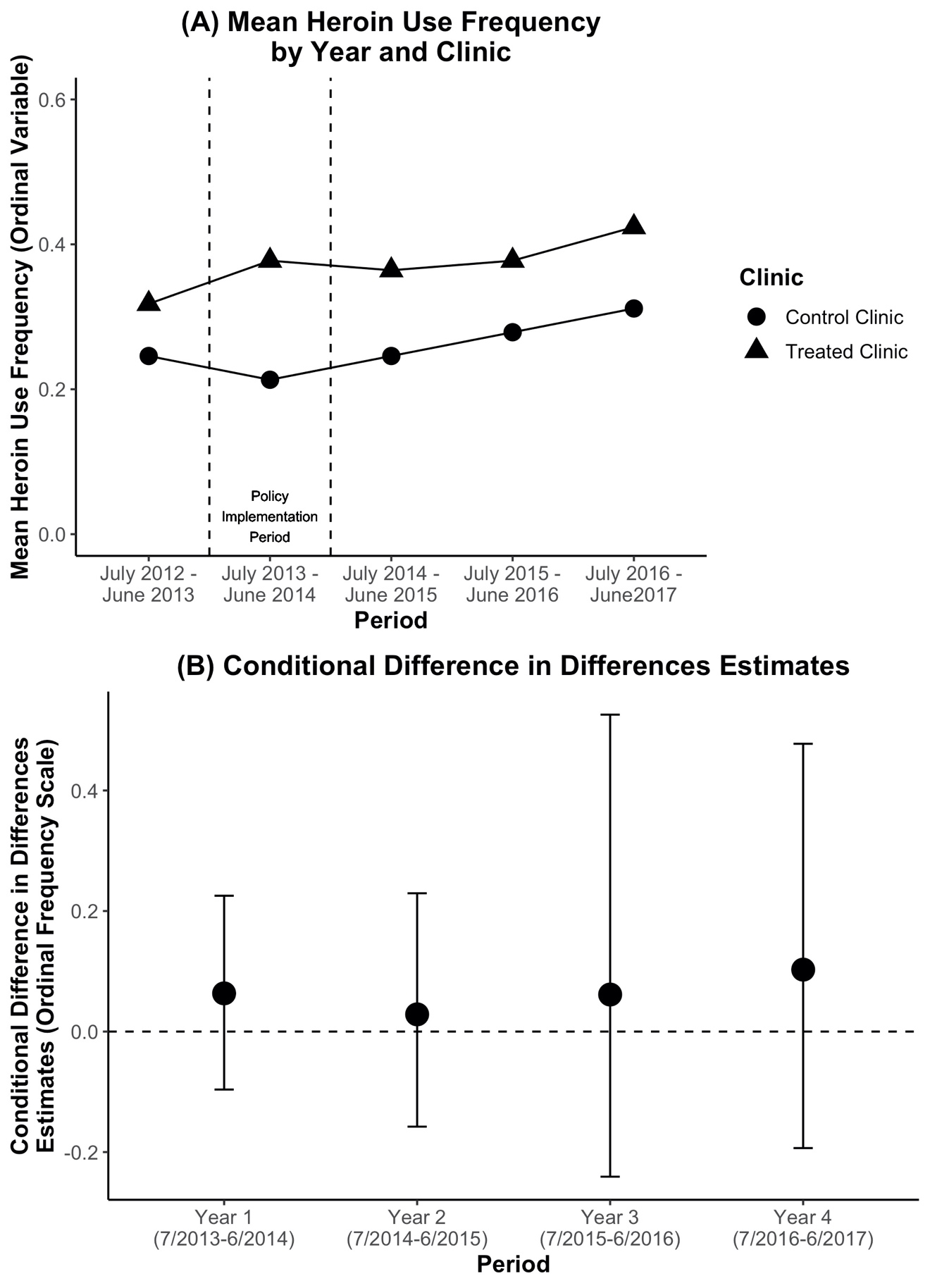
**

**Supplemental Figure 5: (A) Outcome trends by clinic and (B) conditional difference in differences estimates for the any non-prescribed opioid analgesic use outcome**

**
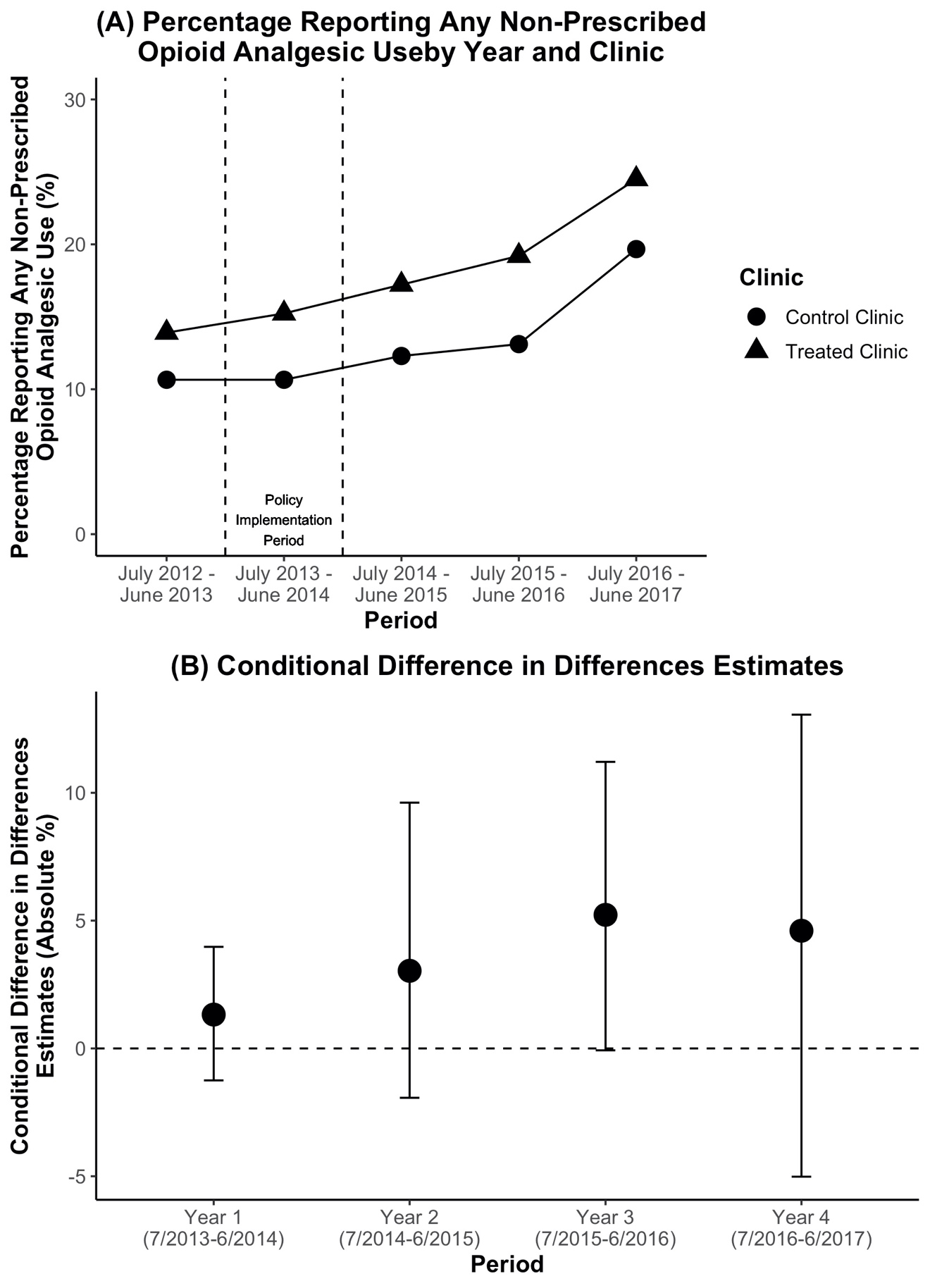
**

**Supplemental Figure 6: (A) Outcome trends by clinic and (B) conditional difference in differences estimates for the non-prescribed opioid analgesic use frequency outcome**

**
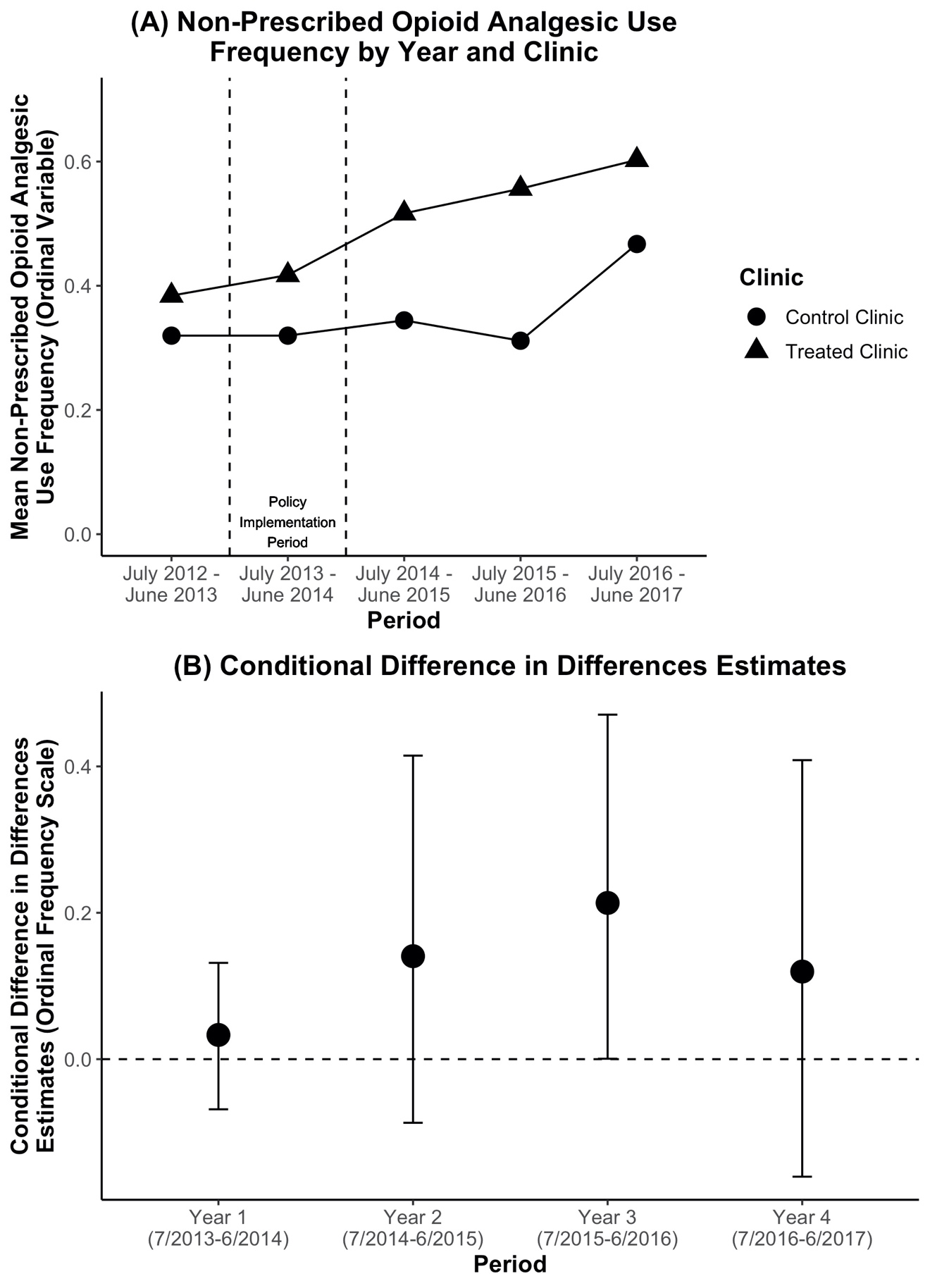
**

**APPENDIX B – Opioid Refill Policy (see PDF)**

**APPENDIX C – Urine Toxicology Policy (see PDF)**

**APPENDIX D – Yellow Flag Committee Referral Form (see PDF)**
