# Supplementary material for: Evaluating the Effects of Opioid Prescribing Policies on Patient Outcomes in a Safety-net Primary Care Clinic
Source: J Gen Intern Med. 2021 Jun 25;37(1):117–24. doi: 10.1007/s11606-021-06920-4 (PMC8738839; doi:10.1007/s11606-021-06920-4)
Supplement: Supplementary file 2 — (PDF 690 kb) [file 11606_2021_6920_MOESM2_ESM.pdf]

[redacted] Clinic Policy/Procedure

Subject: Opioid Refills

Effective Date: 1 July 2013

I. PURPOSE:

The purpose of this policy and procedure is to define a uniform approach to opioid refills, so as to clarify expectations and improve safety for patients and staff, reduce risks and harms from opioids, and reduce staff anxiety.

II. POLICY:

It is the policy of [redacted] that all providers and staff will follow a uniform approach when refilling opioid medications, both during scheduled appointments and when patients drop in, when patients are early or are late for refills, or report lost or stolen opioid medications or prescriptions. All schedules of opioid medications (II-V) are covered by this policy.

III. BACKGROUND:

The primary goal of chronic pain management is safe and effective treatment that optimizes pain control and functional status while minimizing side effects and risks/harms.

The management of chronic pain with controlled substances at [redacted] is complicated by the high prevalence of co-occurring substance use, mental health issues, and/or other psychosocial stressors among individuals with chronic pain. These factors increase the likelihood of concerning behaviors (also called "yellow flag" behaviors) and risks/harms of opioids. Providers and staff should implement a rational, consistent, non-judgmental approach to monitoring for and responding to concerning behaviors related to opioid refills. (NOTE: Concerning or "yellow flag" behaviors are also called aberrant drug-related behaviors in other policies and documents.)

Concerning behaviors are defined in [redacted] and listed at the end of this policy.

IV. PROCEDURE:

A. Patients will get opioid refills by appointment with either the primary medical provider or nurse.
   a. Provider Visits:
      i. Stable patients without any concerning behaviors or "Yellow Flag" notes in the past 12 months are eligible to get up to 3 months worth of opioid prescriptions at one visit. All prescriptions must have the current date, but any extra prescriptions to be filled in the future should have noted on the prescription "fill on/after [date]." The patient should be encouraged to take all prescriptions to the pharmacy at the time received (to avoid lost prescriptions). (In general, pharmacies can hold extra prescriptions. If not, the Walgreens at [redacted] will accept extra prescriptions.)
      ii. Stable patients who have co-morbidities necessitating more frequent provider assessment may have more frequent provider visits.
      iii. Patients who have asked for more than 1 early refill of opioid medications in the past 12 months or who need closer monitoring after other concerning behaviors will have more frequent provider visits (every 1-2 weeks) initially, with decreasing frequency over time if indicated. If concerning behaviors continue, the patient will be referred to the yellow flag committee or the opioids discontinued.

b. Nurse Visits:
    i. Patients may be seen for refills by a nurse but must be seen at least every three months by their provider. If there is not a provider appointment available at three months, then the next available appointment will be scheduled.
    ii. Patients who are starting an opioid trial (who are either opioid naïve or are re-starting opioids) will need frequent monitoring of the efficacy and safety of the regimen, and can be seen by a nurse for some of these visits.
    iii. Patients who need increased monitoring after 1 early refill or other concerning behaviors can be seen by a nurse (see below).
    iv. Patients who have co-morbidities necessitating more frequent assessment but no "Yellow Flag" notes in the past 12 months may be seen by a nurse for opioid refill.
    v. If a concerning behavior is discovered during a nurse visit, medical provider consultation is indicated.

B. Patients who request early opioid refills because of lost or stolen opioid medications or prescriptions are allowed no more than one early refill per 12 month period.
  a. If the patient is dropping in, the triage nurse will alert the patient's team nurse who will see the patient.
  b. The "Yellow Flag" notes (see below) will be checked.
    i. If this is the 1st report of lost or stolen opioid medications or prescriptions within the past 12 months, the incident will be documented in a "Yellow Flag" note. The nurse will remind the patient of pertinent sections of the patient provider agreement or ask the patient to read/sign the informed consent and patient provider agreement for controlled substances if not already done. The patient's provider or provider of the day will write a prescription to last until the next primary care visit (up to a one month supply of opioids).
    ii. If this is the 2nd report of lost or stolen opioid medications or prescriptions within the past 12 months and the situation is uncomplicated (i.e. there is no other reason the patient needs to drop in to see the primary provider or provider of the day), the patient will be seen by the nurse.
      1. The nurse will:
        a. Assess the situation.
        b. Ask the patient to do urine toxicology that day.
        c. Remind the patient of pertinent sections of the patient provider agreement or ask the patient to read/sign the informed consent and patient provider agreement for controlled substances if not already done.
        d. Arrange for the patient to be scheduled to see the primary provider within the next week to have the treatment plan re-assessed. If the primary provider does not have an open appointment, the patient will be scheduled to see a provider on the same team. If no appointments are available within the next week, the patient will be scheduled with the next available provider on the team.
      2. No refill will be given.

C. Patients who request early opioid refills because of running out early must have an assessment to determine the reason/diagnosis and a plan both for increased monitoring and opioid refill if indicated.
    a. If the patient is dropping in, the triage nurse will alert the patient's team nurse. The team nurse will determine whether a provider or a nurse visit is indicated.
    b. If an early refill request occurs during a regularly scheduled appointment with the primary provider, he/she will assess the situation (see below).
    c. Procedure for early opioid refill requests:
        i. The "Yellow Flag" notes (see below) will be checked.
        ii. If this is the 1st request for an early refill and there are no other "Yellow Flag" notes in the last 12 months, and the situation is uncomplicated, (i.e. there is no other reason the patient needs to drop-in to see the primary provider or provider of the day), the patient will be seen by the nurse.
            1. The nurse will :
                a. Assess the situation, including how opioids are being taken, when the last dose of opioids was taken, any use of unprescribed opioids, and any use of illicit substances.
                b. Ask the patient to do urine toxicology that day.
                c. Remind the patient of pertinent sections of the patient provider agreement or ask the patient to read/sign the informed consent and patient provider agreement for controlled substances if not already done.
                d. Arrange for the patient to be scheduled to see the primary provider within the next week to have the treatment plan re-assessed. If the primary provider does not have an open appointment, the patient will be scheduled to see a provider on the same team. If no appointments are available within the next week, the patient will be scheduled with the next available provider on the team.
            2. The nurse will ask the primary provider or provider of the day to write a prescription for opioids to last 1 week, or longer depending on appointment availability.
        iii. If this is the 2nd request for an early refill within 12 months, and the situation is uncomplicated (i.e. there is no other reason the patient needs to drop in to see the primary provider or provider of the day), the patient will be seen by the nurse.
            1. The nurse will:
                a. Assess the situation, including how opioids are being taken, when the last dose of opioids was taken, any use of unprescribed opioids, and any use of illicit substances.
                b. Ask the patient to do urine toxicology that day.
                c. Remind the patient of pertinent sections of the patient provider agreement or ask the patient to read/sign the informed consent and patient provider agreement for controlled substances if not already done.
                d. Arrange for the patient to be scheduled to see the primary provider within the next week to have the treatment plan re-

assessed. If the primary provider does not have an open appointment, the patient will be scheduled to see a provider on the same team. If no appointments are available within the next week, the patient will be scheduled with the next available provider on the team.

2. No refill will be given.

D. Patients who are late for opioid refills (because of a missed appointment or for some other reason) must have an assessment to determine the reason/diagnosis and a plan both for increased monitoring and opioid refill and dose modification if indicated.
   a. If the patient is dropping in, the triage nurse will alert the patient's team nurse. The team nurse will determine whether a provider or a nurse visit is indicated.
   b. If a patient late for opioid refill is seen during a regularly scheduled appointment with the primary provider, he/she will assess situation (see below).
   c. Procedure for late refill:
      i. The "Yellow Flag" notes (see below) will be checked.
      ii. If the patient is less than 1 week late, there are no other "Yellow Flag" notes in the past 12 months, and the situation is uncomplicated, (i.e. there is no other reason the patient needs to drop-in to see primary provider or provider of the day), the patient will be seen by the nurse.
         1. The patient will be reminded of pertinent sections of the patient provider agreement or asked to read/sign the informed consent and patient provider agreement for controlled substances if not already done.
         2. The nurse will ask the primary provider or provider of the day to write a prescription until the next primary provider visit.
      iii. If the patient is more than 1 week late, or there are other "Yellow Flag" notes in the past 12 months, the patient will be seen by the primary provider or provider of the day.

E. All concerning behaviors (as defined by [redacted]) will be documented and tracked using a "Yellow Flag" note in [redacted].
   d. The provider or nurse who sees the patient will write an [redacted] note entitled "Yellow Flag" (as opposed to standard primary care note entitled "Primary Care.") Past [redacted] notes will be checked for previous "Yellow Flag" notes, which will be copied and pasted within the current "Yellow Flag" note. This will allow all concerning behaviors to be easily found and tracked. The note will include the current concerning behavior, the total number of "Yellow Flag" notes, and a standard SOAP note (subjective, objective, assessment, plan) as relevant to chronic pain management.

F. There will be no chronic opioid refills at [redacted]. Any exceptions to this will be discussed with the Medical Director or designee.

G. This policy will be reviewed three months following the effective date or sooner if needed.

List of concerning behaviors taken from [redacted]:

· Requests for refill of controlled substances earlier than expected
· Requests for refill of controlled substances later than expected

· Requests for repeated dose escalations beyond an initial 3 month treatment period
· Requests for a specific/brand name controlled substance/dose
· Report of lost, stolen, damaged prescriptions/medications
· Missing appointments with provider
· Presenting to clinic intoxicated or under the influence of drugs
· Presenting to clinic with signs or symptoms of withdrawal
· History of overdose of controlled substances
· Not adhering to the treatment plan, including adjuvant therapies, diagnostic tests and specialty consultations
· Request for pill count is refused or there is a pill count discrepancy
· Toxicology screening is refused or altered
· Toxicology screening indicates that prescribed medications are absent
· Toxicology screening demonstrates illicit drug use
· Toxicology screening demonstrates use of non-prescribed controlled substances
· Obtaining controlled substances from another provider
· Abusive or threatening behavior towards staff
· Physical violence toward staff
· Altering or stealing a prescription
· Declining functional status despite appropriate therapy
· Arrest for selling prescription controlled substances
· Controlled substance dose reduction in a hospital/other supervised setting due to oversedation

[redacted] Opioid Refill Policy

Patient requests an early or late refill of their opioid medication.

Check patient chart: is the refill early or late?

EARLY: *lost/stolen medication or Rx*

Does the patient's record show any lost or stolen opioid medication or Rx reported in the past 12 months?

NO

- <u>Assess</u> the situation.
- Remind patient of pertinent sections of the <u>patient provider agreement</u> OR (if not already done) ask patient to read & sign informed consent and patient provider agreement for controlled substance.
- <u>Schedule patient</u> to see his/her PCP (or provider on same team) in one month.
- ***Ask PCP or POD to <u>write an Rx</u> for a supply of opioids to last until next PCP visit, up to one month.***

YES

- ***<u>No refill</u> will be given to the patient that day.***

- <u>Assess</u> the situation
- Ask the patient to do a <u>UTOX</u> that day.
- Remind patient of pertinent sections of the <u>patient provider agreement</u> OR (if not already done) ask patient to read & sign informed consent and patient provider agreement for controlled substance.
- <u>Schedule patient</u> to see PCP (or provider on same team) within the next week, or next available appointment with a provider on the team if none available within the week, to re-assess treatment plan.

EARLY: *ran out of medication*

Does the patient's record show any other early opioid refills in the past 12 months?

NO

- <u>Assess</u> the situation, including how opioids are being taken, when the last dose of opioids was taken, any use of unprescribed opioids, and any use of illicit drugs.
- Ask patient to do a <u>UTOX</u> that day.
- Remind patient of pertinent sections of the <u>patient provider agreement</u> OR (if not already done) ask patient to read & sign informed consent and patient provider agreement for controlled substance.
- <u>Schedule patient</u> to see PCP (or provider on same team) within the next week, or next available appointment with a provider on the team if none available within the week, to re-assess treatment plan.
- ***Ask PCP or POD to <u>write an Rx</u> to last 1 week, or longer depending on appointment availability.***

YES

- ***<u>No refill</u> will be given that day.***

- <u>Assess</u> the situation, including how opioids are being taken, when the last dose of opioids was taken, any use of unprescribed opioids, and any use of illicit drugs.
- Ask patient to do a <u>UTOX</u> that day.
- Remind patient of pertinent sections of the <u>patient provider agreement</u> OR (if not already done) ask patient to read & sign informed consent and patient provider agreement for controlled substance.
- <u>Schedule patient</u> to see PCP (or provider on same team) within the next week , or next available appointment with a provider on the team if none available within the week, to re-assess treatment plan.

LATE

1) Does the patient s record have any concerning behavior or "yellow flag" notes in the past 12 months?
2) Is the patient >1 week past the planned refill?
3) Does the patient need to see a provider for any other reason that day?

NO to all questions

- <u>Assess</u> the situation.
- Remind patient of pertinent sections of the <u>patient provider agreement</u> OR (if not already done) ask patient to read & sign informed consent and patient provider agreement for controlled substance.
- ***Ask PCP or POD to <u>write an Rx</u> to last until the next PCP visit.***

YES to one or more question

- ***Patient must be assessed by provider.***

- **Document incident with a "yellow flag" note in the patient's record.**

[redacted] Pain Workgroup
07/01/2013
