# Supplementary material for: Evaluating the Effects of Opioid Prescribing Policies on Patient Outcomes in a Safety-net Primary Care Clinic
Source: J Gen Intern Med. 2021 Jun 25;37(1):117–24. doi: 10.1007/s11606-021-06920-4 (PMC8738839; doi:10.1007/s11606-021-06920-4)
Supplement: Supplementary file 3 — (PDF 1072 kb) [file 11606_2021_6920_MOESM3_ESM.pdf]

[redacted]

[redacted] **Clinic Policy/Procedure**

Subject: Urine Toxicology

Effective Date: 1 July 2014

I. PURPOSE:

The purpose of this policy and procedure is to define a uniform approach for requesting urine toxicology tests for patient who are prescribed opioids and interpreting and acting upon results, so as to ensure consistency and improve safety for patients and staff and reduce risks and harms from opioids.

II. POLICY:

It is the policy of [redacted] that all providers and staff will follow a uniform approach when requesting urine samples from patients who are prescribed opioids for toxicology, interpreting results and taking action based on outcomes. All schedules of opioid medications (II-V) are covered by this policy.

III. BACKGROUND:

The primary goal of chronic pain management is safe and effective treatment that optimizes pain control and functional status while minimizing side effects and risks/harms. In particular, with the reports of increasing rates of death due to accidental overdose from prescription opioids nationally, attention must be paid to those patients who have an elevated risk. Patients are at a higher risk for unintentional opioid overdose with: personal or family history of substance use disorder, history of major mental illness, higher doses of opioids, or use of opioids and benzodiazepines. Patients are at higher risk of problem opioid use with: history of alcohol or drug abuse or dependence, tobacco use, younger age, history of mood disorder, history of childhood sexual abuse, family history of substance abuse, or history of drug-related legal problems (e.g. DUI).

Providers and staff should implement a rational, consistent, non-judgmental approach towards interpreting and responding to urine toxicology results. Urine toxicology

results that suggest that the patient does not have the prescribed medication in their system or that the patient has unprescribed controlled substances or illicit substances are concerning for potential harm due to increased risk for opioid overdose/death, opioid or other substance abuse, or opioid diversion. Although a diagnosis of substance use disorder cannot be made solely using urine toxicology testing results, results can contribute information to make this determination. Patients with an active substance use disorder are poor candidates for treatment with opioids.

IV. PROCEDURE:

A. All patients prescribed chronic opioids will be asked to submit a urine sample for toxicology testing on or before initiating therapy and at least once every 12 months while continuing therapy.
    a. Patients with concerning behaviors or other risk factors may be tested more often at the discretion of the provider.
    b. Patients must provide urine toxicology tests on the day requested. Refusal by the patient to submit a urine sample should be documented as a "Yellow Flag Tracking" note in the [redacted], and the provider may decide not to write the prescription.
    c. If a patient is unable to produce urine (e.g. patients with anuria or neurogenic bladder), or if a patient is mobility-impaired and requires maximal assistance to give a urine sample, providers may use pill counts as a way to monitor patient use of prescribed medications.

B. Results of the urine toxicology test will be interpreted based on the tool provided in the Appendix and information obtained at the time of collection and at the time of discussion of results.

C. If a urine test is inconsistent with the prescribed medication and/or the patient reports concerning behavior, the provider or nurse will document the result/incident, the interpretation, her/his response, and the rationale for the response in a "Yellow Flag Tracking" note.
    a. "Yellow Flag Tracking" notes should be updated when the concerning urine toxicology results are received, so that the plan is clear to any member of the healthcare team when the patient returns.
    b. Response plans will follow the guidelines in the figure and below.
    c. The nurse or provider may request that the primary care provider, team nurse, team member, or another provider (as appropriate) join her/him in communicating any change in treatment plan with the patient. The patient may also be offered the option to discuss the treatment plan with their primary care provider at a later visit, if their provider is not available at that time.

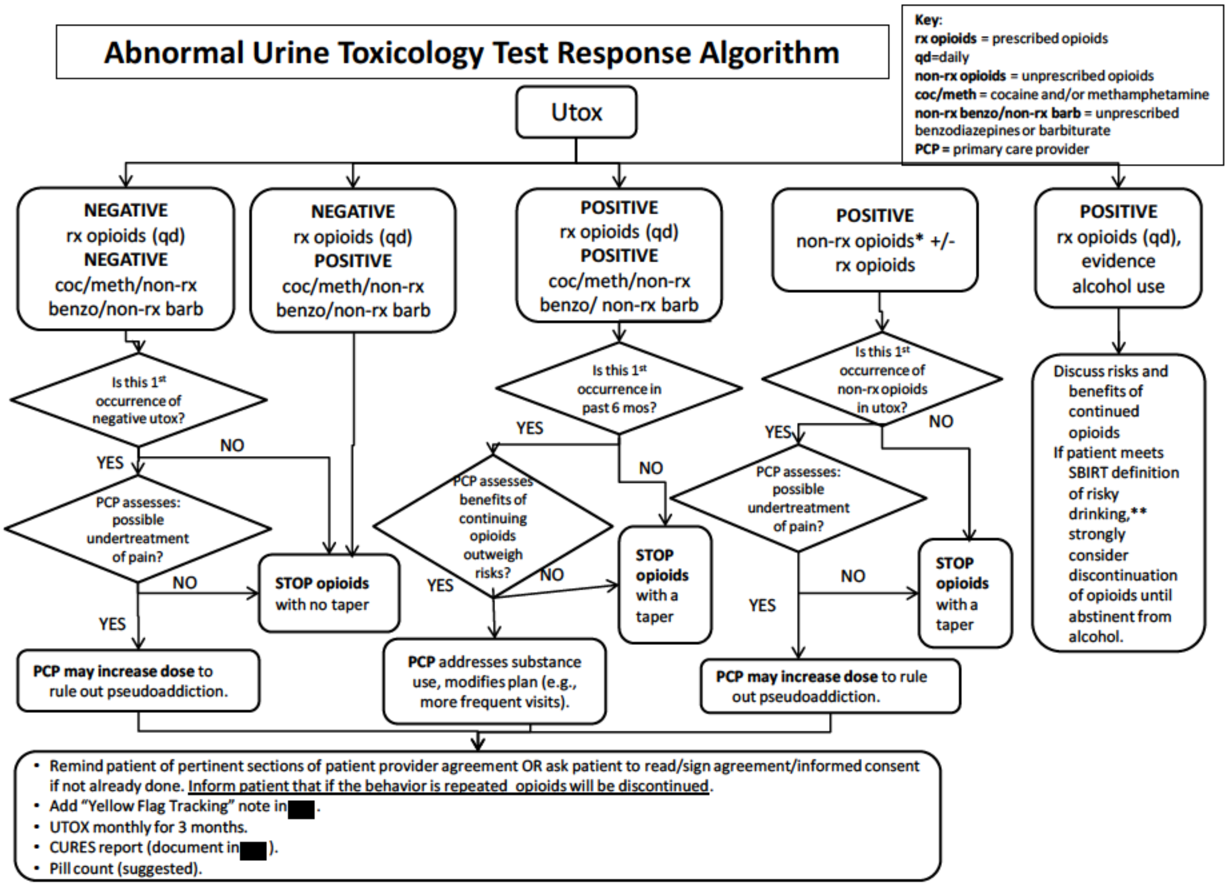

* If unprescribed methadone is present, determine source; if source is methadone treatment program, see text.
** See appendix for definition of risky drinking.

D. Abnormal Urine Toxicology Test Response Procedures

a. **Urine is missing prescribed opioid medication** (applies only to medications dosed daily) and **does not** contain cocaine, methamphetamine, or unprescribed benzodiazepines or barbiturates:

i. The provider should assess the patient for undertreated pain leading to running out of medication early. This is pseudoaddiction.

1. If the provider assesses that pseudoaddiction is the cause of the missing prescribed medication, the provider may increase the dose.
2. If the provider assesses that pseudoaddiction is not cause, the provider should **stop prescribing opioids without a taper.**

ii. If the provider continues the prescribed medication:

1. The provider or nurse must communicate their concern to the patient and remind the patient of pertinent sections of the patient provider agreement or ask the patient to read/sign the informed

consent and patient provider agreement for controlled substances if not already done. **The patient will be informed that if the concerning behavior is repeated, their opioids will be discontinued.**

2. The behavior must be documented in a "Yellow Flag Tracking" note.
3. Urine toxicology testing must be performed at least monthly for 3 months.
4. A CURES report must be obtained and documented in the "Yellow Flag Tracking" note.
5. A pill count is suggested.

iii. If the provider previously responded to a similar result by increasing the opioid dose and **a second urine is missing the prescribed medications, opioids should be discontinued** without a taper. If the opioids are not discontinued, it is the provider's responsibility to bring the case to the Yellow Flag Committee.

b. **Urine is missing prescribed medication** (applies only to medications dosed daily) and **does** contain illicit substances (e.g. cocaine, methamphetamine) or unprescribed benzodiazepines or barbiturates:

i. The provider should **stop prescribing opioids without a taper.**

c. **Urine contains prescribed opioid medication and illicit substances (** e.g. cocaine, methamphetamine) or unprescribed benzodiazepines or barbiturates.

i. The provider may decide to continue prescribing opioids if there have been no similar concerning behaviors in the prior 6 months, and if the benefits of continuing opioids outweigh the risks, for example if the patient is waiting to enter residential treatment. If the provider continues the prescribed medication:

1. The patient's treatment plan will be modified to address substance use, including more frequent visits.
2. The provider or nurse must communicate their concern to the patient and remind the patient of pertinent sections of the patient provider agreement or ask the patient to read/sign the informed consent and patient provider agreement for controlled substances if not already done. **The patient will be informed that if the concerning behavior is repeated, their opioids will be discontinued.**

3. The behavior must be documented in a “Yellow Flag Tracking” note.
4. Urine toxicology testing must be performed at least monthly for 3 months.
5. A CURES report must be obtained and documented in the “Yellow Flag Tracking” note.
6. A pill count is suggested.

ii. Otherwise, the provider **should stop prescribing opioids with a taper**.

iii. If **non-opioid controlled illicit substances are found in the urine a second time within 6 months, prescribing should be discontinued,** with a taper if the urine contains the prescribed medication at the time of discontinuation**.** If an exception is made, it is the provider’s responsibility to bring the case to the Yellow Flag Committee.

d. Urine contains **unprescribed opioids** (with or without prescribed opioids)

i. If unprescribed methadone is present, the provider should determine the source. If not prescribed by a methadone treatment program, follow the procedure below.

1. If the patient is in a methadone treatment program, opioids will be discontinued or tapered until care can be coordinated with the methadone treatment program and a new treatment plan developed. If the patient does not give consent to contact the methadone treatment program, opioids will be discontinued or tapered.

ii. The provider should assess for under-treatment of pain and may consider increasing the dose or discontinuing opioids depending on the assessment. If opioids are continued:

1. The provider or nurse must communicate their concern to the patient and remind the patient of pertinent sections of the patient provider agreement or ask the patient to read/sign the informed consent and patient provider agreement for controlled substances if not already done. The patient will be informed that if the concerning behavior is repeated, their opioids will be discontinued.
2. The behavior must be documented in a “Yellow Flag Tracking” note.

3. A urine toxicology test should be checked at least monthly for 3 months.
4. A CURES report must be obtained and documented in the "Yellow Flag Tracking" note.
5. A pill count is suggested.

ii. If the provider previously responded to a similar result by increasing the opioid dose and **a second urine contains unprescribed opioids,** opioid medications should be discontinued with a taper.

e. Evidence that there is **active alcohol use** by the patient

i. The provider should discuss the risks and benefits of continuing opioids with the patient.

ii. If the patient meets the SBIRT definition of risky drinking (see Appendix), the provider should strongly consider discontinuing opioids until the patient commits to abstinence from alcohol.

iii. The behavior must be documented in a "Yellow Flag Tracking" note.

E. If a provider has discontinued or is tapering opioids due to substance abuse (whether it be opioid, alcohol, illicit drugs, or benzodiazepines), there must be documentation of treatment and/or abstinence for a period of at least 3 months before an opioid re-start will be considered by the provider. Except for alcohol, abstinence will be documented with urine toxicology tests (at least 3 tests completed within 3 months).

a. During this time, patients will continue to be seen to monitor other non-opioid and/or non-pharmacological pain treatments.

b. For patients in residential treatment, the time frame may be shortened.

c. If opioids are restarted, a urine tox test will be obtained at least monthly for 3 months.

d. If at any time during the first 3 months after restarting opioids there are illicit substances or unprescribed controlled substances in the urine, opioids will be discontinued or tapered.

F. If opioids are discontinued a second time, it is the provider's responsibility to bring the case to the Yellow Flag Committee prior to restarting opioids.

APPENDIX: URINE TOXICOLOGY INTERPRETATION GRID

DRUG	POSITIVE TEST	DURATION POSITIVE (IN URINE)	MAY CAUSE FALSE POSITIVE	COMMENT
Amphetamine	Amphetamine	2-4 days	Bupropion<br>Selegiline<br>Trazodone<br>Phentermine<br>Sildenafil<br>Aripiprazole	Confirmation will be negative for all of the false positives except selegiline.<br><br>Some of the designer drugs/bath salts can cross-react.
Methamphetamine	Amphetamine<br>Methamphetamine			
MDMA	MDMA<br>DMA			
Barbiturates	Barbiturates	Usually 1-3 days (phenobarb – 2 weeks)		
Benzodiazepines, e.g.:<br>Clonazepam (Klonopin)<br>Diazepam (Valium)<br>Lorazepam (Ativan)<br>Chlordiazepoxide (Librium)	Benzodiazepine	1-3 days<br>up to 6 wks with heavy use<br>2-4 days for clonazepam		Prone to false negatives.
Buprenorphine				Not detected in standard test. BUP urine test must be ordered specifically.
Cocaine	Cocaine	2-3 days;<br>10-22 days with heavy use	Quinine at very high concentration	
Codeine	Morphine<br>Codeine<br>Hydrocodone	2-3 days		Only patients taking high doses of codeine will test positive for hydrocodone.
Fentanyl				Not detected in standard test. [redacted] lab new test for this must be requested specifically.
Heroin	Morphine<br>Codeine	2-3 days (morphine and codeine)		The only way to distinguish heroin from other opioids is to test for 6-MAM, which [redacted] lab can test for separately
Hydrocodone (e.g., Vicodin)	Hydrocodone<br>Hydromorphone	2-3 days		
Hydromorphone (e.g., Dilaudid)	Hydromorphone	2-3 days		
Methadone	Methadone Metab.	2-4 days		
Morphine	Morphine<br>Hydromorphone	2-3 days		
Oxycodone (e.g., Percocet)	Oxycodone<br>Oxymorphone	1-3 days		Use results for oxycodone assay rather than opiate immunoassay (see detailed note below).

- For the opiate immunoassay, naloxone can cause a false positive.
- The opiate immunoassay has a low cross-reactivity with oxycodone & patients on oxycodone will only screen positive for opiates if they are on a high dose. As a result, the confirmation for codeine, morphine, hydrocodone & hydromorphone will be negative. *The laboratory routinely performs a separate oxycodone assay to provide a more sensitive assay for the presence of oxycodone*.
- For help interpreting results, contact [redacted] or the [redacted] toxicology fellows using the information below. They have access to more detailed test results and can help interpret the outcome, for example if you are concerned about a particular compound causing a false positive or if you want to see a sample testing negative might have had low levels of medication or metabolites.

[redacted]

Interpretation of Urine Drug Testing, [redacted] Toxicology [illegible]

Opiate metabolism

Opiate Confirmation

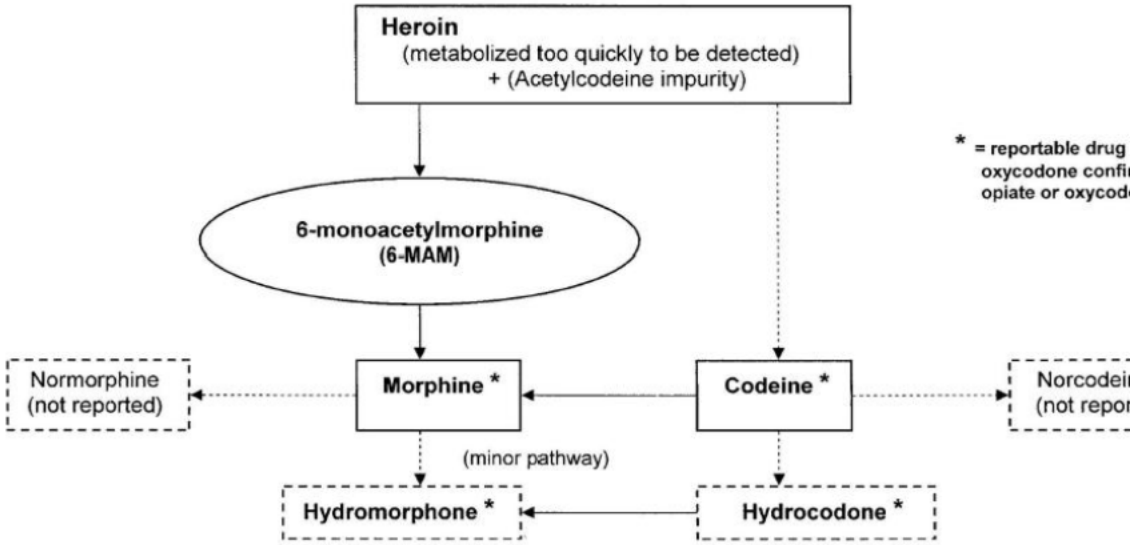

* = reportable drug [illegible]
oxycodone confi[illegible]
opiate or oxycod[illegible]

Oxycodone Confirmation

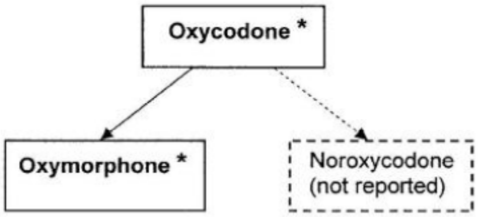

- After **Heroin** use, urine may be positive for morphine onl[illegible] morphine.
- After **Codeine** use, urine may be positive for codeine onl[illegible] (metabolite), or morphine only. Hydrocodone is a trace [illegible] found when very high codeine concentrations are preser[illegible]
- After **Hydrocodone** use, urine may be positive for hydro[illegible] and hydromorphone (metabolite), or hydromorphone onl[illegible]
- After **Hydromorphone** use, urine should be positive onl[illegible]
- After **Morphine** use, urine may be positive for morphine, [illegible] morphine and hydromorphone. Hydromorphone is a trac[illegible] found only when very high morphine concentrations are [illegible]
- After **Oxycodone** use, urine may be positive for oxycod[illegible] oxymorphone (metabolite), or oxymorphone only.

Accessed from [redacted] Lab Manual 07/2013: [redacted]

[redacted]

From “A Pocket Guide for Alcohol Screening and Brief Intervention,” Update 2005 Edition, http://pubs.niaaa.nih.gov/publications/Practitioner/PocketGuide/pocket.pdf

HOW TO SCREEN FOR HEAVY DRINKING

STEP 1 Ask About Alcohol Use

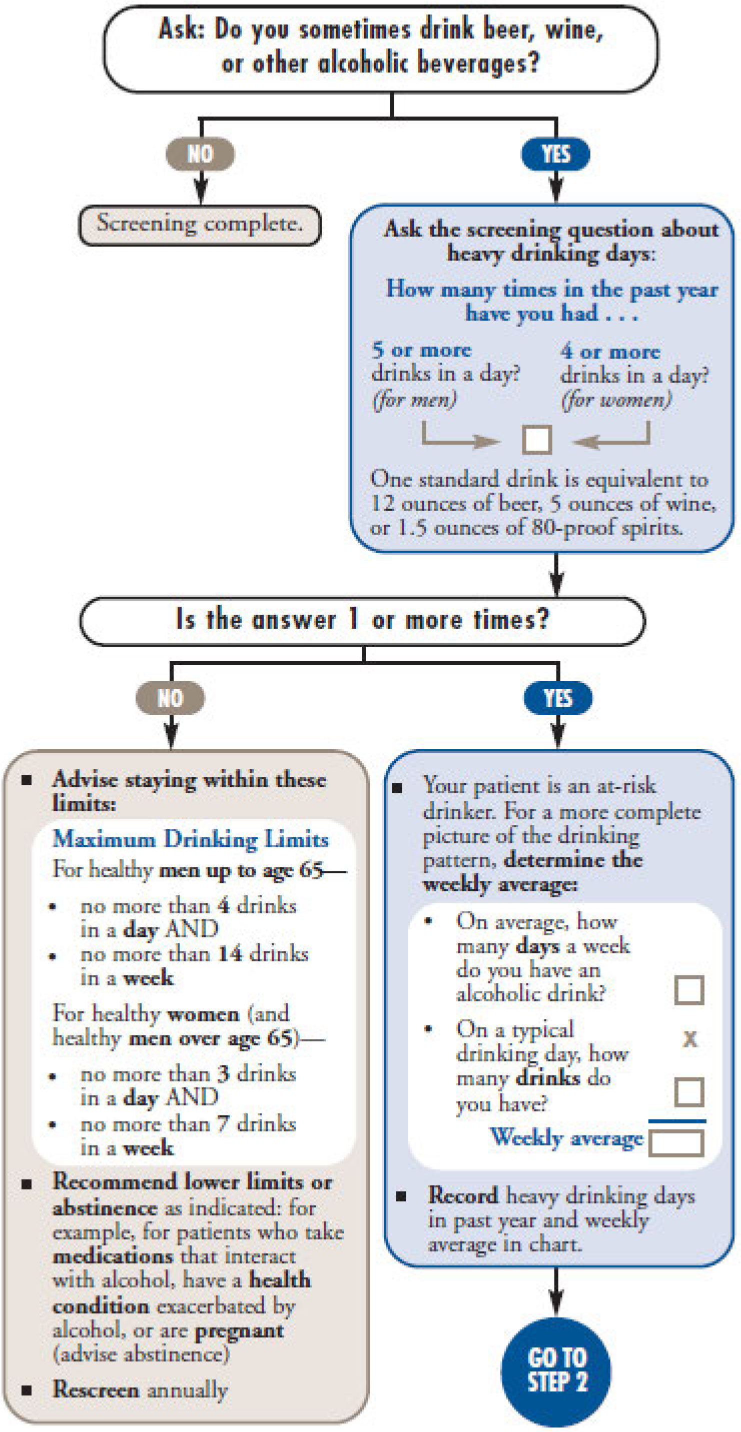

[redacted]

WHAT'S A STANDARD DRINK?

A standard drink in the United States is any drink that contains about 14 grams of pure alcohol (about 0.6 fluid ounces or 1.2 tablespoons). Below are U.S. standard drink equivalents as well as the number of standard drinks in different container sizes for each beverage. These are approximate, since different brands and types of beverages vary in their actual alcohol content.

STANDARD DRINK EQUIVALENTS	APPROXIMATE NUMBER OF STANDARD DRINKS IN:
**BEER or COOLER**	
12 oz. 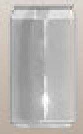 5% alcohol	• 12 oz. = 1<br>• 16 oz. = 1.3<br>• 22 oz. = 2<br>• 40 oz. = 3.3
**MALT LIQUOR**	
8–9 oz. 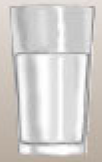 7% alcohol	• 12 oz. = 1.5<br>• 16 oz. = 2<br>• 22 oz. = 2.5<br>• 40 oz. = 4.5
**TABLE WINE**	
5 oz. 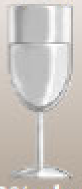 12% alcohol	• a 750-mL (25-oz.) bottle = 5
**80-proof SPIRITS (hard liquor)**	
1.5 oz. 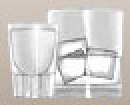 40% alcohol	• a mixed drink = 1 or more*<br>• a pint (16 oz.) = 11<br>• a fifth (25 oz.) = 17<br>• 1.75 L (59 oz.) = 39<br><br>**Note:* Depending on factors such as the type of spirits and the recipe, one mixed drink can contain from one to three or more standard drinks.

[redacted]
