# Supplementary material for: Evaluating the Effects of Opioid Prescribing Policies on Patient Outcomes in a Safety-net Primary Care Clinic
Source: J Gen Intern Med. 2021 Jun 25;37(1):117–24. doi: 10.1007/s11606-021-06920-4 (PMC8738839; doi:10.1007/s11606-021-06920-4)
Supplement: Supplementary file 4 — (PDF 197 kb) [file 11606_2021_6920_MOESM4_ESM.pdf]

Yellow Flag Committee Referral Form

Patient Name: (enter here) MRN: (enter here) DOB: MM/DD/YYYY

PCP: (enter here) Patient Provider Agreement/Informed Consent signed: (select) Date: MM/DD/YYYY

What is your reason for referral? (Can use check boxes on p2.)

Concerning (aberrant medication related) behaviors

Please list all concerning behaviors. (Can use check boxes on p2.)

Latest urine drug screening: MM/DD/YYYY

Did results show prescribed medications? ☐ Yes ☐ No
Did results show unprescribed medications? ☐ Yes ☐ No
Did results show illicit substances? ☐ Yes ☐ No

History of prior incongruous urine drug screens? ☐ Yes ☐ No
Please attach CURES report to referral form.

Contributing Factors for Opioid Abuse/Diversion/Overdose

History of substance abuse/addiction, including nicotine and alcohol	(select)
Family history of substance abuse/addiction	(select)
ADHD, ADD, PTSD, Schizophrenia, Bipolar disorder, OCD	(select)
Depression	(select)
History of oversedation with medication	(select)
History of overdose	(select)
History of physical or sexual abuse	(select)

Pain Management History

Diagnosis/cause of pain: (enter here)
(Required) Current medication list, especially opioids, up to date in [redacted]? (select)

Is the patient on methadone maintenance? (select) If so, what dose? (enter here)

Has pain improved with opioid therapy? (select)
If unknown, please explain: (enter here)

Has function improved with opioid therapy? (select)
If unknown, please explain: (enter here)

Has quality of life improved with opioid therapy? (select)
If unknown, please explain: (enter here)

What non-opioid approaches to pain has the patient tried (eg. medications, ice/heat, injections, surgery, PT/physical activity, activity pacing, stress/anger management/coping, cognitive behavioral therapy, pain group, psychotherapy, massage, acupuncture, chiropractic, meditation/mindfulness, yoga)? (enter here)

Mental health diagnosis? (select) Treatment? (select)
Provide details: (enter here)

Active substance use disorder? (select) Treatment? (select)
Provide details: (enter here)

Active alcohol use disorder? (select) Treatment? (select)

Provide details: (enter here)

Psychosocial situation (e.g. housing, benefits, relationships/support) (enter here)

Reason(s) for Referral (check all that apply)

☐ Patients on opioids, stimulants, or benzodiazepines whose use has become problematic for the patient, provider, or staff.
☐ Patients with 3 or more yellow flags (see above).
☐ Patients with a behavioral agreement for disruptive or violent behavior related to opioids.
☐ Patients on opioids who are at higher risk for overdose or death:
  - ☐ Patients with overdose in the past 12 months.
  - ☐ Patients on high dose opioids. (>400mg morphine/d; >100mg methadone/d, excluding methadone maintenance).

Yellow Flags (check all that apply)

List of concerning behaviors taken from [redacted] policy [redacted]:

☐ Requests for refill of controlled substances earlier than expected
☐ Requests for refill of controlled substances later than expected
☐ Requests for repeated dose escalations beyond an initial 3 month treatment period
☐ Requests for a specific/brand name controlled substance/dose
☐ Report of lost, stolen, damaged prescriptions/medications
☐ Missing appointments with provider
☐ Presenting to clinic intoxicated or under the influence of drugs
☐ Presenting to clinic with signs or symptoms of withdrawal
☐ History of overdose of controlled substances
☐ Not adhering to the treatment plan, including adjuvant therapies, diagnostic tests and specialty consultations
☐ Request for pill count is refused or there is a pill count discrepancy
☐ Toxicology screening is refused or altered
☐ Toxicology screening indicates that prescribed medications are absent
☐ Toxicology screening demonstrates illicit drug use
☐ Toxicology screening demonstrates use of non-prescribed controlled substances
☐ Obtaining controlled substances from another provider
☐ Abusive or threatening behavior towards staff
☐ Physical violence toward staff
☐ Altering or stealing a prescription
☐ Declining functional status despite appropriate therapy
☐ Arrest for selling prescription controlled substances
☐ Controlled substance dose reduction in a hospital/other supervised setting due to oversedation
